# Positive feedback regulation of p53 transactivity by DNA damage-induced ISG15 modification

**DOI:** 10.1038/ncomms12513

**Published:** 2016-08-22

**Authors:** Jong Ho Park, Seung Wook Yang, Jung Mi Park, Seung Hyeun Ka, Ji-Hoon Kim, Young-Yun Kong, Young Joo Jeon, Jae Hong Seol, Chin Ha Chung

**Affiliations:** 1 School of Biological Sciences, College of Natural Sciences, Seoul National University, Seoul 08826, Korea; 2 Department of Biochemistry, Institute of Medical Science, Chungnam National University School of Medicine, Daejon 34134, Korea; 3 The 2nd Division of Natural Sciences, National Academy of Science, Seoul 06579, Korea

## Abstract

p53 plays a pivotal role in tumour suppression under stresses, such as DNA damage. ISG15 has been implicated in the control of tumorigenesis. Intriguingly, the expression of ISG15, UBE1L and UBCH8 is induced by DNA-damaging agents, such as ultraviolet and doxorubicin, which are known to induce p53. Here, we show that the genes encoding ISG15, UBE1L, UBCH8 and EFP, have the p53-responsive elements and their expression is induced in a p53-dependent fashion under DNA damage conditions. Furthermore, DNA damage induces ISG15 conjugation to p53 and this modification markedly enhances the binding of p53 to the promoters of its target genes (for example, *CDKN1* and *BAX*) as well as of its own gene by promoting phosphorylation and acetylation, leading to suppression of cell growth and tumorigenesis. These findings establish a novel feedback circuit between p53 and ISG15-conjugating system for positive regulation of the tumour suppressive function of p53 under DNA damage conditions.

## Abstract (web summary)

The ‘genome guardian' p53 has a well-established role in suppressing tumour development after DNA damage. Here the authors show that expression of the ubiquitin-like protein ISG15 is regulated by p53 which in turn is modified by ISG15 to enhance binding to target gene promoters.

## Graphical abstract

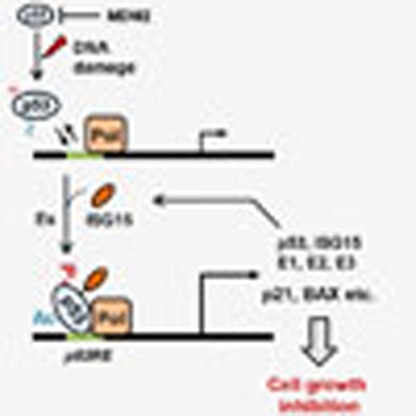

The p53 tumour suppressor has been regarded as ‘guardian of the genome'^1^ or ‘cellular gatekeeper'^2^, as it coordinates cellular responses to various stress signals, such as DNA damage, abnormal oncogene activation, telomere erosion and hypoxia^3^,^4^. Under normal conditions, p53 is downregulated by several ubiquitin E3 ligases, including the major MDM2 ligase, and subsequent degradation by proteasome. Notably, the expression of MDM2 is induced by p53, thus forming a negative feedback loop for keeping p53 at a low level^5^,^6^,^7^. Under stressed conditions, however, p53 is stabilized and activated by disruption of its interaction with MDM2 and the other negative regulators through phosphorylation and acetylation. The activated p53 then binds to a specific DNA sequence, called the p53-responsive element (*p53RE*), for transcriptional activation of its target genes (for example, *CDKN1, BAX* and *PUMA*) that mediate cell cycle arrest and apoptosis^8^,^9^,^10^.

Since p53 is involved in the control of numerous critical cellular processes, its transactivity needs to be tightly regulated^11^. The p53 activity is regulated by a wide variety of post-translational modifications, including the modification by ubiquitin-like proteins, in addition to phosphorylation, methylation, acetylation and ubiquitination. MDM2- and FBXO11-mediated neddylation inhibits p53 transcriptional activity^12^,^13^, whereas sumoylation promotes it^14^,^15^. Recently, it has been reported that ISG15, the product of the interferon (IFN)-stimulated gene 15, can be conjugated primarily to misfolded p53 and this modification promotes the degradation of p53 by proteasome^16^. However, it remains unknown when and how the modification of p53 by ubiquitin-like proteins occurs for the control of the p53 activity.

ISG15 is the first reported ubiquitin-like protein^17^. ISG15 expression is robustly induced by type-I IFNs, lipopolysaccharides and viral infection^18^,^19^. Like ubiquitination, protein ISGylation is catalysed by a three-step enzyme system: UBE1L as an ISG15-activating E1 enzyme, UBCH8 as an ISG15-conjugating E2 enzyme and EFP and HERC5 as ISG15 E3 ligases^19^,^20^,^21^,^22^. This protein ISGylation can be reversed by an ISG15-deconjugating enzyme, UBP43 also called USP18 (ref. 23). In addition to conjugation to target proteins, type-I IFN-induced ISG15 is secreted from leukocytes, such as monocytes and lymphocytes, and serves as a cytokine that stimulates synthesis and secretion of IFN-γ^24^,^25^.

Numerous studies using murine system have demonstrated that protein modification by ISG15 mediates anti-viral responses. Mice lacking Ube1L exhibit increased susceptibility to influenza B virus infection^26^ and ISG15-deficient mice are more susceptible to influenza A and B, Sindbis and herpes virus infections^27^. In addition, a loss-of-function mutation within the *Usp18* gene (*Usp18*^*lty9*^) in mice confers increased susceptibility to *Salmonella* Typhimurium^28^. On the other hand, in human, free ISG15 secreted from granulocytes plays a crucial role as an IFN-γ-inducing cytokine for optimal antimycobacterial immunity^29^,^30^, while intracellular ISG15 functions in UBP43-mediated downregulation of type-I IFN signalling and prevention of type-I IFN-dependent auto-inflammation^31^. Remarkably, ISG15 deficiency in human, unlike in mice, causes little or no change in susceptibility to viral infection^29^,^30^, indicating distinctive as well as diverse roles of ISG15 in mammals.

Protein ISGylation and de-ISGylation appear to also function in the control of DNA damage responses. We have recently shown that DNA-damaging agents, such as doxorubicin, induce ISGylation of the oncogenic ΔNp63α protein for suppression of epithelial tumours^32^. We also have shown that ultraviolet induces ISGylation of PCNA for termination of DNA damage-induced error-prone translesion synthesis for maintaining genome stability^33^. Notably, during these studies, we found that the expression of ISG15, UBE1L and UBCH8 can be induced by DNA-damaging agents, such as ultraviolet, doxorubicin and camptothecin, all of which are known to induce p53. These findings raised a possibility that DNA damage-induced expression of the ISG15-conjugating machinery is under the control of p53.

In the present study, we show that all of the genes encoding ISG15, UBE1L, UBCH8 and EFP have *p53RE*s in the promoter regions for their p53-mediated expression under DNA damage conditions. Remarkably, p53 serves as a target for ISGylation and this modification dramatically increased the binding of p53 to the promoter regions of its target genes as well as of its own gene, forming a positive feedback loop, which would amplify the expression of the genes for cell cycle arrest and apoptosis, such as *CDKN1* and *BAX*. Collectively, these results indicate that ISGylation of p53 plays a critical role in cell growth inhibition and thereby in suppression of tumour development under DNA damage conditions.

## Results

### p53 induces the expression of ISG15-conjugating system

**Figure 1:**
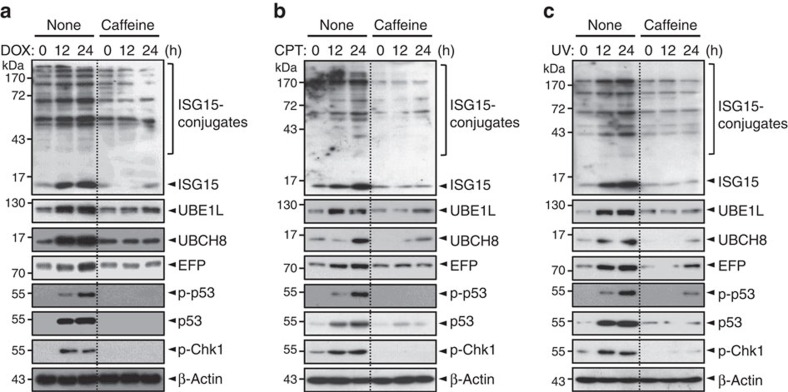
ATM/ATR kinases induce the expression of ISG15-conjugating system. *p53*^*+/+*^ HCT116 cells were treated with 1 μM doxorubicin (DOX) (**a**), 0.25 μM camptothecin (CPT) (**b**) and 30 J m^−2^ ultraviolet (UV) (**c**). Immediately after the treatment, the cells were incubated with 5 mM caffeine for increasing periods. The cells were washed with iced PBS, and lysed in 50 mM Tris-HCl (pH 7.4), 150 mM NaCl, 1 mM EDTA, 1 mM NEM, 1 mM sodium orthovanadate, 1 mM NaF, 1 mM PMSF, 0.2% (v/v) Triton X-100 and 1 X protease inhibitor cocktail. The samples were centrifuged for 30 min at 16,000*g* and the supernatant fractions (cell lysates) were subjected to immunoblot with anti-ISG15, anti-UBE1L anti-UBCH8, anti-EFP, anti-phospho-p53 (p-p53), anti-p53, anti-phospho-Chk1 (p-Chk1) and anti-β-actin antibodies (from the top to the bottom, respectively).

We have recently shown that DNA-damaging agents, such as doxorubicin, camptothecin or ultraviolet, induce the expression of both messenger RNA (mRNA) and protein levels of ISG15, UBE1L and UBCH8 (refs 32, 33). These findings suggest that the expression of ISG15, UBE1L and UBCH8 may be regulated by p53 via DNA damage-induced activation of ATM and ATR kinases. To test this possibility, *p53*^*+/+*^ HCT116 cells (human colon carcinoma) were incubated with caffeine, an ATR and ATM inhibitor^34^, immediately after treatment with the DNA-damaging agents (Fig. 1). As expected, caffeine abrogated phosphorylation of Chk1 and p53 and thereby the expression of p53. Remarkably, the drug also strongly inhibited the expression of EFP as well as of ISG15, UBE1L and UBCH8 (all together henceforth referred to as ISG15-conjugating system). Supplementary Figure 1 shows the quantitative data for the changes in the levels of ISG15-conjugating system in the presence and absence of caffeine under DNA damage conditions. Note that we examined the effect of caffeine on EFP expression, since of two known ISG15 E3 ligases, EFP, but not HERC5, interacts with p53 for ISGylation (see below). Similar results were obtained when caffeine was treated to other cancer cells, including U2OS (human osteosarcoma), MCF7 (human breast carcinoma) and A549 (human lung carcinoma), all of which are known to express normal p53 (Supplementary Fig. 2). One exception, however, was HeLa cells (human cervical carcinoma): caffeine showed relatively little effect on the expression of ISG15, although it inhibited that of UBE1L, UBCH8 and EFP (Supplementary Fig. 3). These results suggest that the expression of ISG15-conjugating system is regulated by p53, although it remains unclear how DNA damage-induced expression of ISG15 in HeLa cells could occur in the presence of caffeine.

To verify further p53-mediated induction of ISG15-conjugating system, we compared their expression in *p53*^*+/+*^ and *p53*^*−/−*^ cells. Ultraviolet treatment to *p53*^*−/−*^ cells showed little or no effect on the expression of ISG15-conjugating system, unlike that to *p53*^*+/+*^ cells (Supplementary Fig. 4a,b). Furthermore, the expression of a p53-specific short hairpin RNA (shRNA; shp53), but not a nonspecific shRNA (shNS), in *p53*^*+/+*^ cells abrogated ultraviolet-induced expression of ISG15-conjugating system (Supplementary Fig. 4c,d). Similar results were obtained when doxorubicin or camptothecin was treated in place of ultraviolet. Taken together, these results indicate that the expression of ISG15-conjugating system is upregulated by p53 under DNA damage conditions.

### The genes encoding ISG15-conjugating system has *p53REs*

We next examined whether the genes encoding ISG15-conjugating system have the p53-response elements (*p53RE*s) for their p53-mediated expression. Sequence analysis showed the presence of several candidate sequences (termed *RE1* to *RE4*) in the promoter regions of the *ISG15, UBE1L, UBCH8* and *EFP* genes (Supplementary Fig. 5), which are similar to the known sequence of *p53RE*: RRRCWWGYYY-X_n_-RRRCWWGYYY, of which R stands for purine, W for adenine or thymine, Y for pyrimidine and X for spacer with *n*∼21 (ref. 35).

**Figure 2:**
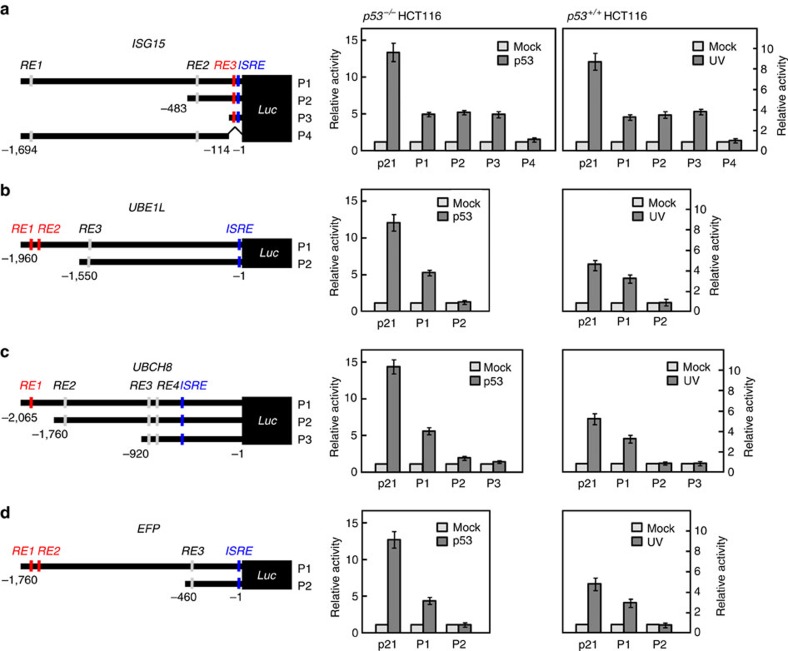
*p53RE*s in the promoter regions of the genes for ISG15-conjugating system. Deletions of the promoter regions in the *ISG15* (**a**), *UBE1L* (**b**), *UBCH8* (**c**) and *EFP* genes (**d**) were fused to a *Luc* reporter vector (pGL3) (left panels). The resulting vectors (P1–P4) were transfected to *p53*^*−/−*^ HCT116 cells with pcDNA-HA-p53. *p53*^+/+^ HCT116 cells transfected with the same P1–P4 vectors were treated with and without ultraviolet (UV). *p21-Luc* was expressed in the cells as a control. These cells were then subjected to assay for the luciferase activity (right panels). The activity seen without any expression or treatment was expressed as 1.0 and the others were expressed as its relative values. Error bar, ±s.d. (*n*=3).

To identify true *p53RE*(s) among the *RE*s, serial deletions were generated and fused to a luciferase (*Luc*) reporter vector (Fig. 2, left panels). The resulting vectors (termed P1 to P4) were expressed in *p53*^*−/−*^ HCT116 cells with and without p53. The *p53*^*+/+*^ HCT116 cells were also transfected with P1–P4, followed by treatment with and without ultraviolet. These cells were then subjected to assay for the luciferase activity. The cells transfected with P1–P3 having *RE3* of the *ISG15* gene, P1 having *RE1/RE2* of *UBE1L*, P1 having *RE1* of *UBCH8*, and P1 having *RE1/RE2* of *EFP*, but not with the other vectors, showed a significant increase in the luciferase activity upon p53 expression or ultraviolet exposure (Fig. 2, right panels). Furthermore, ChIP analysis revealed that overexpressed p53 in *p53*^*−/−*^ HCT116 cells effectively binds to each of the identified *p53RE*s of the genes, as it does to *p53RE* of the *p21* gene (Supplementary Fig. 6). These results indicate that the promotor regions of the *ISG15*, *UBE1L*, *UBCH8* and *EFP* genes have *p53RE*s and their expressions are positively regulated by p53.

### IFN-independent expression of ISG15-conjugating system

**Figure 3:**
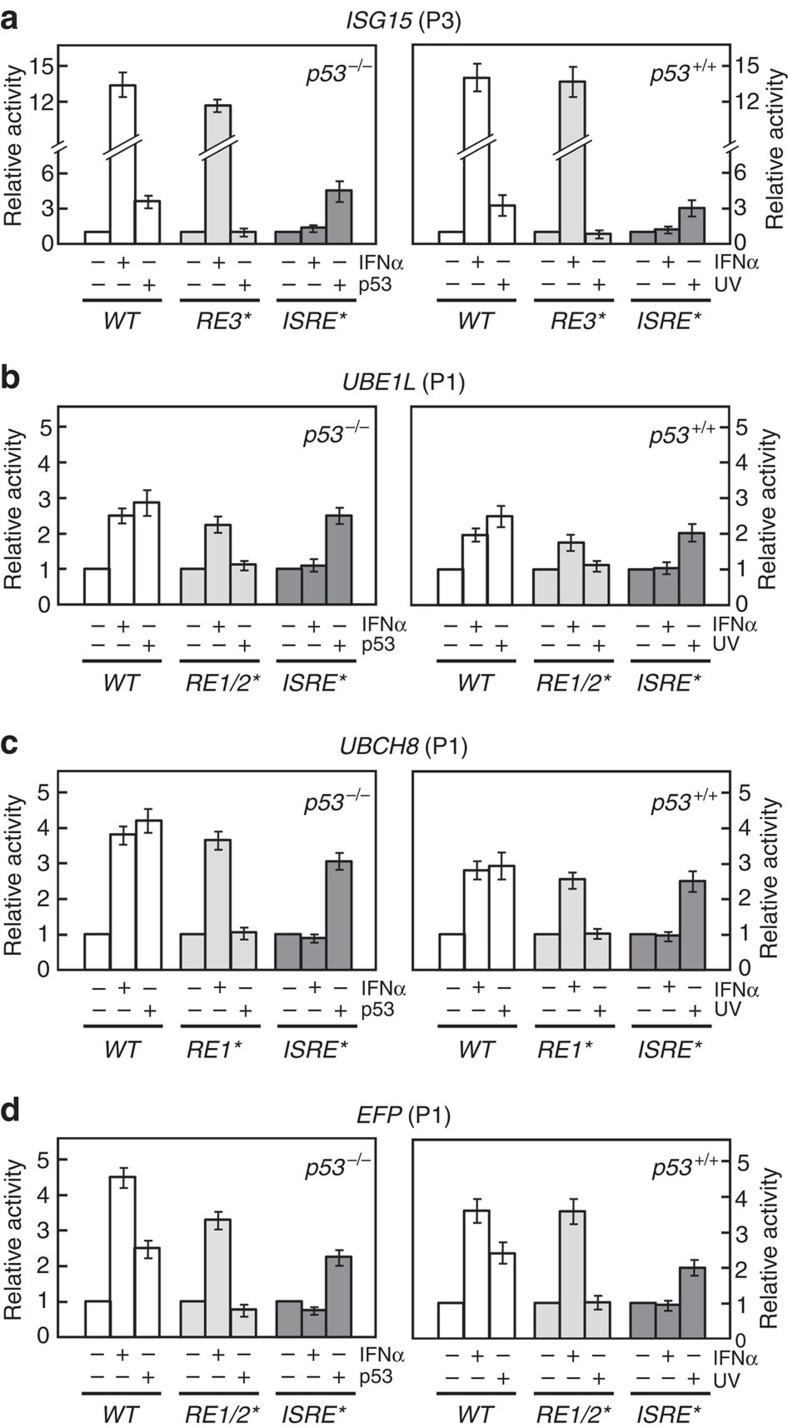
p53-induced expression of ISG15-conjugating system is independent of type-I IFNs. In P1 reporter vectors (see Fig. 2), *RE3* of the *ISG15* gene (**a**), *RE1/RE2* of *UBE1L* (**b**), *RE1* of *UBCH8* (**c**) and *RE1/RE2* of *EFP* (**d**) were mutated as shown in Supplementary Fig. 5 and the mutated *RE*s were marked by the asterisks. *ISRE*s of the genes were also mutated and marked by the asterisks. After transfection of the vectors, p53 were expressed in *p53*^*−/−*^ HCT116 cells or 1,000 U ml^−1^ of interferon-α (IFNα) was treated to the cells (left panels). *p53*^+/+^ HCT116 cells transfected with the same reporter vectors were treated with and without IFNα or ultraviolet (UV; right panels). The cells were then subjected to assay for the luciferase activity. The activity seen without any expression or treatment was expressed as 1.0 and the others were expressed as its relative values. Error bar, ±s.d. (*n*=3).

Type-I IFNs are known to induce the expression of ISG15-conjugating system by activating IFN-stimulated gene factor 3 (ISGF3), which binds to the highly conserved 13-bp sequence of *ISRE* in their promoters (Supplementary Fig. 5)^21^,^22^,^36^,^37^. To determine whether p53 could induce the expression of ISG15-conjugating system independently of type-I IFNs, we generated mutations (marked with red in Supplementary Fig. 5) in *p53RE*s and *ISRE*s of *Luc* reporter vectors for preventing their interaction with p53 and ISGF3, respectively. The mutated vectors (marked by the asterisks in Supplementary Fig. 5) were expressed in *p53*^*−/−*^ HCT116 cells with and without p53 (Fig. 3, left panels). The *p53*^*+/+*^ HCT116 cells were also transfected with the same reporter vectors, followed by treatment with and without ultraviolet (Fig. 3, right panels). The mutation of *p53RE*s abrogated p53- and ultraviolet-mediated expression of ISG15-conjugating system, but not IFNα-mediated expression. On the other hand, the mutation of *ISRE*s prevented IFNα-mediated expression of ISG15-conjugating system, but not p53- and ultraviolet-mediated expression. In addition, ultraviolet markedly increased the level of ISGylated cellular proteins only when *p53*^*−/−*^ HCT116 cells were supplemented with p53, while IFNα increased it regardless of the presence of p53 (Supplementary Fig. 7). These results indicate that p53 and type-I IFNs can independently induce the expression of ISG15-conjugating system.

To confirm further whether p53 induces the expression of ISG15-conjugating system, p53-null H1299 cells were transfected with an empty plasmid or a vector expressing HisMax-p53. After exposure to ultraviolet, the cells were incubated for increasing periods. The levels of ISG15-conjugating system as well as of ISGylated cellular proteins gradually increased after ultraviolet treatment in cells expressing p53, but not in cells lacking p53 regardless of ultraviolet treatment (Supplementary Fig. 8a). Moreover, the mRNA levels of ISG15-conjugating system increased in a p53-dependent manner under the DNA damage condition (Supplementary Fig. 8b), further demonstrating that p53 positively regulates the expression of ISG15-conjugating system.

### DNA damage induces p53 ISGylation

**Figure 4:**
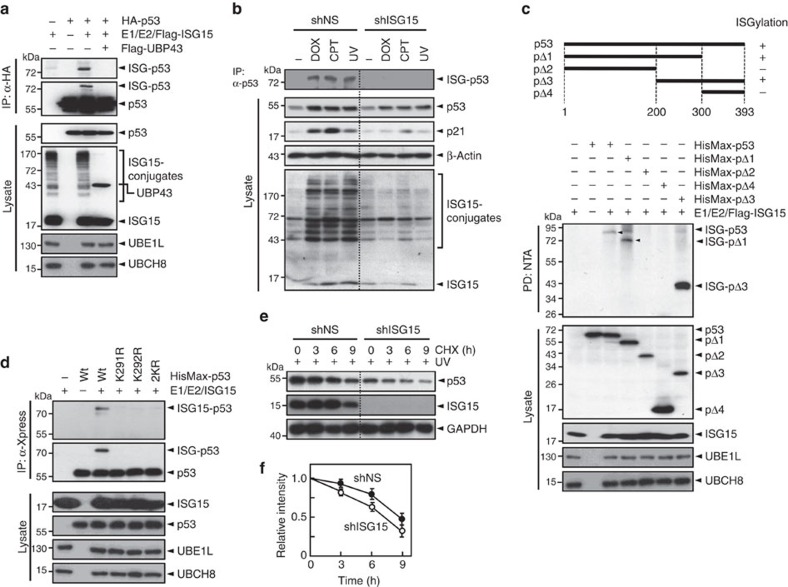
DNA damage induces p53 ISGylation. (**a**) HA-p53 and Flag-ISG15, Myc-UBE1L (E1) and Myc-UBCH8 (E2) were expressed in HEK293T cells with or without Flag-UBP43. The cell lysates were subjected to immunoprecipitation (IP) with anti-HA antibody followed by immunoblot with anti-Flag or anti-HA antibody. They (lysates) were also directly probed with anti-HA, anti-Flag or anti-Myc antibody. (**b**) *p53*^+/+^ HCT116 cells that had been transfected with shNS or shISG15 were treated with doxorubicin (DOX) or camptothecin (CPT) for 24 h. They were also irradiated with ultraviolet (UV), and then incubated for 24 h. The cell lysates were subjected to immunoprecipitation with anti-p53 antibody followed by immunoblot with anti-ISG15 antibody. They were also directly probed with respective antibodies. (**c**) Deletions of p53 (pΔ1–pΔ4) were tagged with HisMax to their N-termini, and expressed in HEK293T cells with Flag-ISG15, Myc-UBE1L and Myc-UBCH8. The cell lysates were subjected to pull-down with NTA resins followed by immunoblot with anti-Flag antibody. (**d**) Wild-type p53 (Wt) or its K-to-R mutants were expressed in HEK293T cells with Flag-ISG15, Myc-UBE1L and Myc-UBCH8. The cell lysates were subjected to immunoprecipitation with anti-Xpress antibody followed by immunoblot with anti-Flag or anti-Xpress antibody. (**e**) HCT116 (*p53*^+/+^) cells were transfected with shNS or shISG15. After exposure to ultraviolet, the cells were subjected to incubation with 0.2 mg ml^−1^ cycloheximide (CHX) for increasing periods followed by immunoblot analysis. (**f**) Experiments in **e** were repeated and the band intensities were scanned by using a densitometer and normalized by those of GAPDH. The normalized densities seen at ‘0' time points were expressed as 1.0 and the others were expressed as its relative values. Error bar, ±s.d. (*n*=3).

p53 induces MDM2 expression, and MDM2 ubiquitinates p53. Since p53 induces the expression of ISG15-conjugating system, we examined whether p53 could be ISGylated in retrospect. Overexpression of ISG15, UBE1L and UBCH8 in HEK293T cells led to the appearance of a slow-migrating band with the size of ∼74 kDa and this band could be disappeared upon co-expression of UBP43, an ISG15-deconjugating enzyme (Fig. 4a), indicating that the band represents ISGylated p53. Furthermore, endogenous p53 could be ISGylated upon exposure of *p53*^*+/+*^ HCT116 cells to doxorubicin, camptothecin and ultraviolet and this modification could be abolished by the expression of an ISG15-specific shRNA (shISG15), but not by that of shNS (Fig. 4b). These results indicate that p53 serves as an endogenous target for ISGylation under DNA damage conditions.

To identify ISG15 acceptor site(s) in p53, various deletions of p53 (termed P1 to P4) were generated and expressed in HEK293T cells with ISG15, UBE1L and UBCH8. P1 (amino acids 1–300) and P3 (201–393), but not the others, were conjugated by ISG15 (Fig. 4c), indicating that the amino acid sequence 201–300 has ISGylation site(s). Since the 201–300 sequence has two lysine residues (Lys291 and Lys292), each and both of them were replaced by arginine in full-length p53 by site-directed mutagenesis. The K-to-R mutation of each (K291R or K292R) and both Lys residues (2KR) completely abrogated p53 ISGylation (Fig. 4d), indicating that both Lys291 and Lys292 act as the major ISG15 acceptor sites in p53, although it remains unclear how the mutation of one Lys residue influences ISGylation of the other Lys residue.

To determine whether ISGylation of p53 influences its stability under DNA damage conditions, wild-type p53 and 2KR were expressed in *p53*^*−/−*^ HCT116 cells. The cells were treated with and without ultraviolet, and then incubated with cycloheximide for increasing periods. Without ultraviolet treatment, the stability of 2KR is significantly higher than that of wild-type p53 (Supplementary Fig. 9), consistent with a previous report^38^ showing that both Lys291 and Lys292 serve as the major ubiquitination sites of the MKRN1 ubiquitin E3 ligase, which primarily acts under normal conditions. As expected, ultraviolet treatment led to a dramatic increase in the stability of p53. Notably, however, the 2KR mutation showed little or no effect on ultraviolet-mediated increase in p53 stability, as if ISGylation of p53 does not influence its stability. Since these experiments were performed under overexpression conditions, we examined whether knockdown of ISG15 influences the stability of endogenous p53 under DNA damage conditions. ISG15 depletion led to a significant, although not dramatic, decrease in p53 stability (Fig. 4e,f). Since ISG15 knockdown markedly reduces the expression of p53 itself and its downstream targets, particularly MDM2 (see below), ISG15 depletion-mediated decrease in p53 stability is likely compromised by the reduction in MDM2 expression. Taken together, these results indicate that ISGylation positively regulates the stability of p53 under DNA damage conditions.

### EFP serves as a p53-specific ISG15 E3 ligase

**Figure 5:**
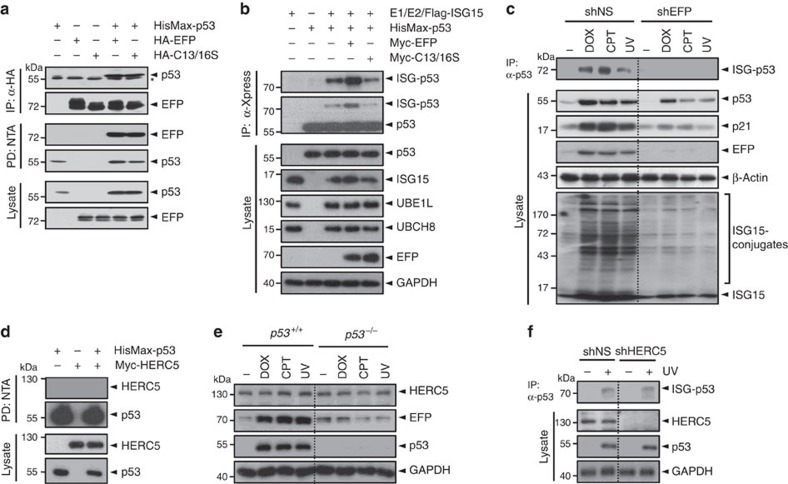
EFP serves as a p53-specific ISG15 E3 ligase. (**a**) HA-EFP or its inactive C13/16S mutant was expressed in HEK293T cells with HisMax-p53. The cell lysates were subjected to immunoprecipitation with anti-HA antibody or pull-down with NTA resins followed by immunoblot with anti-Xpress or anti-HA antibody. The asterisk indicates IgG heavy chain. (**b**) HisMax-p53, Flag-ISG15, Myc-UBE1L (E1) and Myc-UBCH8 (E2) were expressed in HEK293T cells with Myc-tagged EFP or its C13/16S mutant. The cell lysates were subjected to immunoprecipitation with anti-Xpress antibody followed by immunoblot anti-Flag or anti-Xpress antibody. (**c**) *p53*^+/+^ HCT116 cells that had been transfected with shNS or shEFP were treated with doxorubicin or camptothecin for 24 h. They were also treated with ultraviolet (UV), and then incubated for 24 h. The cell lysates were subjected to immunoprecipitation with anti-p53 antibody followed by immunoblot with anti-ISG15 antibody. (**d**) Myc-HERC5 was expressed in HEK293T cells with HisMax-p53. The cell lysates were subjected to pull-down with NTA resins followed by immunoblot with anti-Myc or anti-Xpress antibody. (**e**) After treatment with DNA-damaging agents, the HCT116 cells were subjected to incubation for 24 h followed by immunoblot with anti-HERC5, anti-EFP or anti-p53 antibody. (**f**) *p53*^+/+^ HCT116 cells expressing shNS or shHERC5 were exposed to ultraviolet. After incubation for 24 h, the cells were subjected to immunoprecipitation with anti-p53 followed by immunoblot with anti-ISG15 antibody.

EFP and HERC5 have been identified as the major E3 ligases for ISG15 conjugation^20^,^22^. To identify p53-specific ISG15 E3 ligase among them, we first examined the ability of EFP to interact with p53. Not only wild-type EFP but also its catalytically inactive mutant, of which the active site Cys13 and Cys16 residues were replaced by serine (C13/16S), could interact with p53 (Fig. 5a), indicating that the catalytic activity of EFP is not required for its interaction with p53. In addition, overexpression of EFP, but not its inactive form (C13/16S), markedly increased p53 ISGylation (Fig. 5b). Furthermore, knockdown of EFP by shEFP prevented DNA damage-induced p53 ISGylation (Fig. 5c), indicating that EFP serves as an E3 ligase of p53. In contrast, HERC5 was unable to interact with p53 (Fig. 5d). Moreover, treatment with DNA-damaging agents did not show any effect on HERC5 expression in both *p53*^*+/+*^ and *p53*^*−/−*^ HCT116 cells, unlike that on EFP expression (Fig. 5e). In addition, knockdown of HERC5 showed little or no effect on ultraviolet-induced p53 ISGylation in *p53*^*+/+*^ HCT116 cells (Fig. 5f). These results indicate that neither DNA damage nor p53 influences the expression of HERC5.

To map the regions for the interaction between p53 and EFP, we first examined the ability of p53 deletions (PΔ1 to PΔ4) to interact with EFP. PΔ1 (amino acid 1–300) and PΔ3 (201–393), but not PΔ2 (1–200) and PΔ4 (301–393), could interact with EFP, indicating that EFP-binding site is present in the middle region of p53 (201–300) (Supplementary Fig. 10a). Various deletions of EFP (termed EΔ1 to EΔ4) were also generated and tested for their ability to bind p53. EΔ1 (1–438) and EΔ3 (218–630) were capable of binding to p53, whereas EΔ2 (1–217) and EΔ4 (439–630) could not (Supplementary Fig. 10b). These results indicate that p53-binding site lies in the middle region of EFP (218–438).

### ISGylation of p53 promotes its transactivity

**Figure 6:**
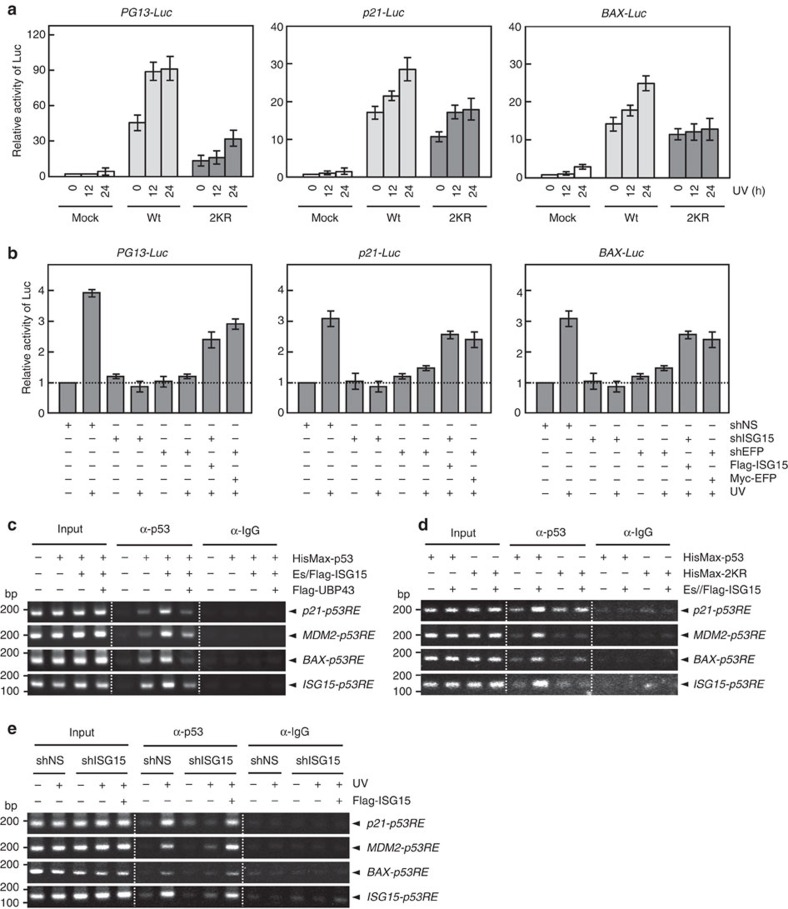
ISGylation promotes p53 transactivity. (**a**) Wild-type p53 (Wt), its 2KR mutant, and an empty vector (Mock) were expressed in H1299 cells with *PG13-Luc, p21-Luc* and *BAX-Luc*. After exposure to ultraviolet (UV), they were incubated for various periods, and subjected to assay for the luciferase activity. Transfection efficiency was normalized by using β-galactosidase constructs. The luciferase activity seen without any treatment (that is, ‘0' time in Mock) was expressed as 1.0 and the others were as its relative values. Error bar, ±s.d. (n=3). (**b**) shNS, shISG15 or shEFP was expressed in *p53*^+/+^ HCT116 cells with and without shRNA-insensitive Flag-ISG15 or Myc-EFP. After exposure to ultraviolet, the cells were incubated for 24 h and subjected to the assay for the luciferase activity as in **a**. Error bar, ±s.d. (n=3). (**c**) HisMax-p53 and ISG15-conjugating system (Es/Flag-ISG15) were expressed in *p53*^*−/−*^ HCT116 cells with and without Flag-UBP43. The cell lysates were subjected to ChIP assay using anti-p53 antibody or anti-mouse IgG. Bound DNAs were subjected to PCR using the probes for *p53RE*s of *CDKN1*, *MDM2*, *BAX* and *ISG15*. (**d**) HisMax-p53 and HisMax-2KR were expressed in *p53*^*−/−*^ HCT116 cells with or without ISG15-conjugating system. The cell lysates were subjected to ChIP as in **c**. (**e**) shNS or shISG15 were expressed in *p53*^+/+^ HCT116 cells with and without shRNA-insensitive Flag-ISG15. After exposure to ultraviolet, the cells were incubated for 24 h, and then subjected to ChIP as in **c**. Note that shISG15 was directed to a 5′-UTR region. **a**, **b** and **e**, similar results were obtained when the cells were treated with doxorubicin or camptothecin in place of ultraviolet.

Of note was the finding that knockdown of ISG15 or EFP results in a significant reduction in p53 expression (see Figs 4b and 5c), raising a possibility that p53 ISGylation might be involved in the control of its transactivity, in addition its stability, and thereby in the expression of its target genes (including its own). To test this possibility, p53 and its ISGylation-defective 2KR mutant were expressed in p53-null H1299 cells that had been transfected with p53-responsive reporter vectors, including *PG13-Luc, p21-Luc* and *BAX-Luc*. The 2KR mutation caused a marked decrease in ultraviolet-induced p53 transactivity (Fig. 6a). Similar results were obtained when doxorubicin was treated to cells (Supplementary Fig. 11). Consistently, prevention of p53 ISGylation by knockdown of ISG15 or EFP also dramatically reduced the p53 activity and this reduction could be reversed by co-expression of shRNA-insensitive ISG15 or EFP (Fig. 6b). Immunoblot data for cells used in Fig. 6a,b were shown in Supplementary Fig. 12a,b, respectively. These results indicate that p53 ISGylation promotes the expression of its target genes as well as of its own gene.

To test a possibility whether ISGylation influences Chk1 phosphorylation and thereby promotes the expression of ISG15-conjugating system, *p53*^*+/+*^ HCT116 cells transfected with shISG15 or shEFP were exposed to ultraviolet. Knockdown of ISG15 or EFP markedly reduced p53 expression, but showed little or no effect on Chk1 phosphorylation (Supplementary Fig. 13). These results indicate that p53 ISGylation positively controls the expression of ISG15-conjugating system without any influence on the activation of its upstream regulators.

In an attempt to determine the mechanism for ISGylation-mediated stimulation of p53 transactivity, we examined the effect of p53 ISGylation on the binding of p53 to the promoters of its target genes. ChIP analysis revealed that the expression of ISG15-conjugating system with p53 in *p53*^*−/−*^ HCT116 cells markedly increases the binding of p53 to *p53RE*s of its target genes, including *p21, MDM2, BAX* and *ISG15*, and this increase could be abrogated by co-expression of UBP43 (Fig. 6c). On the other hand, the expression of ISG15-conjugating system showed little or no effect on the binding of ISGylation-deficient 2KR mutant to *p53RE*s of its target genes (Fig. 6d). Furthermore, knockdown of ISG15 dramatically reduced ultraviolet-induced binding of p53 to the promoter regions but this effect could be reversed upon complementation of a shRNA-insensitive ISG15 (Fig. 6e). Similar results were obtained when experiments in Fig. 6c–e were repeated and the extracted DNAs were subjected to quantitative PCR analysis (Supplementary Fig. 14). These results indicate that p53 ISGylation plays a crucial role in the promotion of p53 binding to the promoters of its target genes under DNA damage conditions.

Acetylation of p53 has been shown to strongly increase its affinity of *p53RE*^39^,^40^. In addition, it has been shown that p53 phosphorylation increases its binding to p300 acetyl-transferase^41^,^42^. To determine whether p53 ISGylation influences its phosphorylation and acetylation, H1299 cells expressing wild-type p53 or its 2KR mutant were exposed to ultraviolet. Immunoblot analysis revealed that the 2KR mutation almost completely abrogates ultraviolet-induced acetylation of p53 (Supplementary Fig. 15a,b). It also significantly inhibited p53 phosphorylation. These results indicate that p53 ISGylation promotes its phosphorylation and acetylation and, in turn, its ability to bind to *p53RE*. These results also raised a possibility that under DNA damage conditions, p53 might be ISGylated, initially by the basal ISG15 and its conjugating system for early activation of p53 by phosphorylation and acetylation and then by belatedly induced ISG15-conjugating system for further potentiation of p53 transactivity. To test this possibility, we examined whether p53 ISGylation occurs before its phosphorylation and acetylation immediately after ultraviolet treatment. The levels of p53, phospho-p53 and acetylated p53 gradually increased in order, whereas ISGylated p53 could not be detected at least for 3 h after ultraviolet treatment (Supplementary Fig. 15c). These results indicate that phosphorylation and acetylation of p53 precede its ISGylation for induction of ISG15-conjugating system as well as of other downstream targets.

### p53 ISGylation suppresses cell growth and tumorigenesis

**Figure 7:**
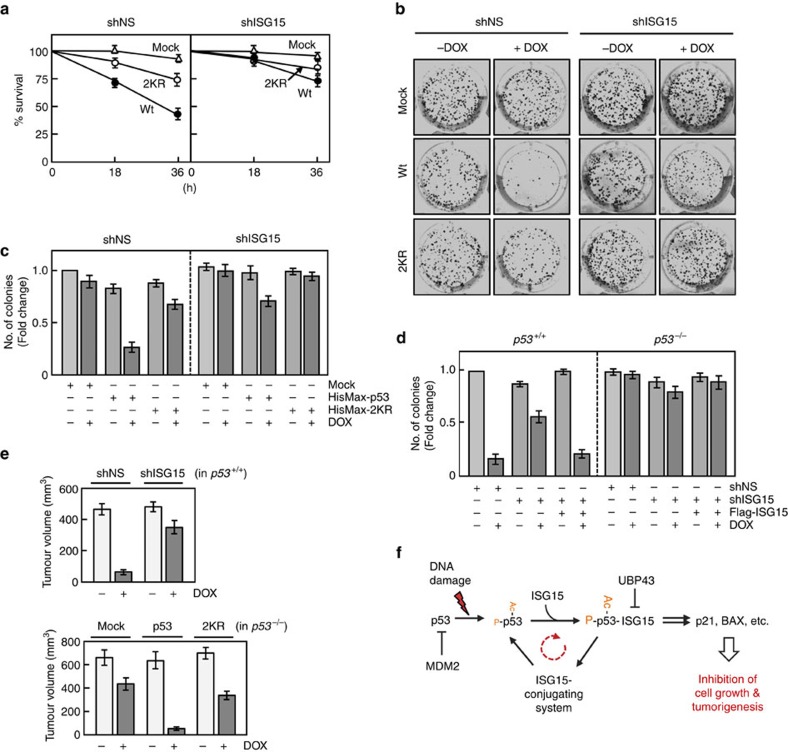
p53 ISGylation suppresses cell growth and tumour development. (**a**) *p53*^*−/−*^ HCT116 cells expressing wild-type p53 (Wt), its 2KR mutant, or an empty vector (Mock) were transfected with shNS or shISG15. After treatment with doxorubicin for increasing periods, viable cells were counted by staining with trypan blue exclusion. Error bar, ±s.d. (*n*=3). (**b**) *p53*^*−/−*^ HCT116 cells prepared as in **a** were incubated with or without doxorubicin for 10 days. The colonies were then stained with crystal violet. (**c**) Experiments were repeated as in **b** and the colonies were counted. Error bar, ±s.d. (*n*=3). (**d**) shNS or shISG15 were expressed in both *p53*^+/+^ and *p53*^*−/−*^ HCT116 cells with and without shRNA-insensitive Flag-ISG15. After treatment with doxorubicin, experiments were then performed as in **b**. Error bar, ±s.d. (*n*=3). (**e**) *p53*^*+/+*^ HCT116 cells expressing shNS or shISG15 (top) and *p53*^*−/−*^ HCT116 cells expressing wild-type p53 or its 2KR mutant (bottom) were injected to BALB/c nude mice. ‘Mock' in the bottom panel indicates the cells transfected with an empty vector. After treatment with PBS or doxorubicin, tumour volumes were determined. Error bar, ±s.d. (*n*=5). (**f**) A model for positive feedback regulation of p53 transactivity by ISGylation under DNA damage conditions. ‘Ac' and ‘P' indicate acetylated and phosphorylated, respectively.

To determine whether p53 ISGylation influences its ability to inhibit cell growth, p53 or its 2KR mutant was expressed in *p53*^*−/−*^ HCT116 cells that had been transfected with shNS or shISG15. Doxorubicin treatment led to a marked decrease in the survival of p53-expressing cells, but showed much less effect on the survival of 2KR-expressing cells, and these inhibitory effects on cell survival could be abrogated by ISG15 knockdown (Fig. 7a). Furthermore, the clonogenic assays revealed that 2KR-expressing cells are much less sensitive to the drug-mediated inhibition of colony formation than wild-type p53-expressing cells and these effects could be abolished by ISG15 knockdown (Fig. 7b,c). Consistently, knockdown of ISG15 markedly reduced doxorubicin-mediated inhibition of colony formation of *p53*^*+/+*^ HCT116 cells and this reduction could be prevented upon co-expression of shRNA-insensitive ISG15 (Fig. 7d). However, ISG15 knockdown regardless of co-expression of shRNA-insensitive ISG15 showed little or no effect on colony formation by *p53*^*−/−*^ HCT116 cells. Immunoblot data for cells used in Fig. 7a,b,d were shown in Supplementary Fig. 16a–c, respectively. Similar results were obtained when cells were treated with camptothecin or ultraviolet in place of doxorubicin. We next examined whether p53 ISGylation promotes apoptosis under DNA damage conditions. *p53*^*−/−*^ HCT116 cells expressing wild-type p53 or its 2KR mutant were treated with different concentrations of doxorubicin. TUNEL assay revealed that apoptosis is markedly reduced in cells expressing 2KR mutant compared with those expressing wild-type p53 (Supplementary Fig. 17). Collectively, these results indicate that DNA damage-induced ISGylation of p53 promotes cell growth inhibition and apoptosis by increasing its transactivity.

To determine whether ISGylation of p53 could consequently promote its tumour suppressive function, an *in vivo* tumorigenesis assay was performed by injecting BALB/c nude mice with *p53*^*+/+*^ HCT116 cells stably expressing shNS or shISG15 and *p53*^*−/−*^ HCT116 cells stably expressing wild-type p53 and its 2KR mutant. Without doxorubicin treatment, all the mice developed large tumours (Fig. 7e and Supplementary Fig. 18). On the drug treatment, tumour volumes were dramatically reduced in mice injected with *p53*^*+/+*^ cells expressing shNS and *p53*^*−/−*^ cells expressing ectopic p53. Consistently, immunoblot analysis of these tumour tissues showed a marked increase in the level of apoptotic BAX protein. In contrast, tumours with appreciable sizes were developed in mice injected with *p53*^*+/+*^ cells expressing shISG15 and *p53*^*−/−*^ cells expressing 2KR mutant, even when doxorubicin was treated, indicating the involvement of p53 ISGylation in tumour suppression. Notably, however, the size of tumour developed in mice injected with *p53*^*−/−*^ cells expressing 2KR mutant is smaller than that in mice *p53*^*−/−*^ cells transfected with an empty vector (Mock) under doxorubicin-treated conditions, indicating that 2KR is still capable of suppressing tumour development by inducing p53 phosphorylation and BAX expression, although much less efficiently than wild-type p53. Collectively, these results indicate that ISGylation of p53 plays a crucial role in promotion of its tumour-suppressive function.

### UBP43 represses expression of ISG15-conjugating system

We have recently shown that ISGylation of PCNA terminates ultraviolet-induced, error-prone translesion DNA synthesis and that the ISGylation process is reversed by UBP43, whose expression is induced at the later periods, for regaining the function of PCNA as a processive factor in normal replication^33^. To determine whether UBP43 expression is also induced at the later periods under different DNA damage conditions, *p53*^*+/+*^ HCT116 cells expressing shNS or shISG15 were treated with doxorubicin for increasing periods. Immunoblot analysis revealed that in shNS-expressing cells, the levels of p53, p-p53, MDM2, p21 and BAX gradually increased until 24–36 h after doxorubicin treatment, and remained elevated (or began to fall) thereafter without further increase (Supplementary Fig. 19, left panels). The levels of ISG15-conjugating system also increased until 24–36 h after the drug treatment, but declined thereafter, concomitantly with a marked increase in the level of UBP43. These results strongly suggest that UBP43 deISGylates p53 at the later period of doxorubicin treatment and thereby downregulates the expression of ISG15-conjugating system for prevention of prolonged promotion of p53 transactivity.

Upon depletion of ISG15, the expression of p53, p-p53, MDM2, p21 and BAX was markedly reduced and delayed, although their levels gradually increased at the later periods after doxorubicin treatment (Supplementary Fig. 19, right panels). In addition, ISG15 knockdown abrogated doxorubicin-induced rise-and-fall of the expression of ISG15-conjugating system, confirming that p53 ISGylation promotes the expression of its downstream targets. Interestingly, however, ISG15 knockdown showed little or no effect on the expression time and level of UBP43, indicating that the expression of UBP43 is not associated with p53 ISGylation. However, it remains unknown how the belated expression of UBP43 is regulated under DNA damage conditions.

## Discussion

In the present study, we showed that the promoters of the *ISG15, UBE1L, UBCH8 and EFP* genes have *p53RE*s for their p53-mediated expression, independent of type-I IFNs. We further demonstrated that p53 serves as a target for conjugation by ISG15 and this modification dramatically stimulates the binding of p53 to *p53RE*s by promoting its phosphorylation and acetylation, indicating a positive feedback regulatory circuit operates for potentiation of p53 transactivity. On the basis of this finding, we propose a model for the role of DNA damage-induced ISGylation in the control of p53 transactivity and, in turn, in inhibition of cell and tumour growth (Fig. 7f). Under normal conditions, the cellular level of p53 is kept low by MDM2-mediated ubiquitination and proteasomal degradation. On exposure to DNA-damaging agents, such as doxorubicin, camptothecin and ultraviolet, the p53 level gets elevated and activated for expression of its downstream targets, including ISG15-conjugating system, and thereby for ISGylation of p53 (as well as of other substrates), albeit initially to a low level. This ISGylation dramatically stimulates phosphorylation and acetylation of p53, which promotes its binding to *p53RE* for more efficient and belated expression of p53 itself and its downstream targets, including p21, BAX and ISG15-conjugating system. Thus, it appears that the sequential phosphorylation, acetylation and ISGylation of p53 forms a cycle with the expression of ISG15-conjugating system for amplified expression of p53 downstream targets. Collectively, the positive feedback regulation of p53 transactivity by ISG15 modification seems to play a crucial role in the expression of its target genes involved in cell growth inhibition and, in turn, in suppression of tumour development under DNA damage conditions. Interestingly, however, the expression of UBP43 is induced at the later periods after treatment with DNA-damaging agents. This increase in UBP43 expression should lead to p53 de-ISGylation for termination of positive feedback control of p53 transactivity, particularly in cells that had already committed to apoptotic cell death as well as in residual cells that survived after DNA repair.

Of note was the finding that ISGylation of p53 leads to a marked increase in its ability to bind *p53RE*s. Previously, it was shown that p53 phosphorylation increases its binding to p300 acetyl-transferase and p53 acetylation dramatically promotes its binding to *p53-RE*^39^,^40^,^41^,^42^. In this study, we found that ultraviolet-induced p53 ISGylation promotes its phosphorylation and acetylation. Thus, it appears likely that ISG15 molecules conjugated to p53 serve as molecular scaffolds that recruit DNA damage-activated protein kinase(s) and acetyl-transferases(s), such as p300, for phosphorylation and acetylation of p53 and thereby for elevating its affinity to *p53REs*.

Recently, Huang *et al*.^16^ have shown that p53 can be ISGylated at multiple unknown sites by overexpression of HERC5, but not by EFP. They also showed that misfolded p53 is preferentially ISGylated for proteasomal degradation and that ISG15 depletion leads to the accumulation of misfolded p53, which then acts dominant negatively on p53 function, such as on apoptosis. This HERC5-mediated ISGylation of p53 is in contrast with our finding that p53 interacts with EFP, but not HERC5, even under overexpression conditions. Moreover, knockdown of EFP, but not HERC5, was found to prevent DNA damage-induced p53 ISGylation (Fig. 5c, f). However, it has been shown that HERC5 is physically associated with polyribosomes, and can modify a wide range of newly synthesized proteins by ISG15 in a co-translational manner^43^. Therefore, it appears possible that polyribosome-associated HERC5 ISGylates newly synthesized, unstructured p53 protein without strong interaction with the E3 ligase.

Previously, we have shown that doxorubicin can induce the expression of ISG15, UBE1L and UBCH8, leading to ISGylation of ΔNp63α, in HNSCC013 and HCC1937 cells, despite the fact that these cells express mutated nonfunctional p53 (refs 44, 45). Thus, it appears that certain cell types are capable of inducing ISG15-conjugating system independently of p53 under DNA damage conditions. It has been reported that ΔNp63α, lacking the amino (N)-terminal transactivation domain, has a second transactivation domain, and therefore can regulate expression of distinct subset of genes^46^,^47^,^48^, although it is better known as p53 inhibitor, owing to the presence of the carboxy (C)-terminal transcriptional inhibition domain^49^,^50^. Alternatively, therefore, ΔNp63α itself might induce ISG15-conjugating system for its own ISGylation.

Protein ISGylation has been implicated in both tumour development and tumour suppression^51^. The level of ISG15 is elevated in many human primary cancers, such as in tumours of bladder, breast, endometrium and prostate^52^,^53^,^54^,^55^,^56^. Recently, knockout of ISG15 was shown to suppress K-ras-induced lung tumour as well as V-Src-mediated tumour formation in mice^57^. In addition, UBP43-deficient mice have been shown to develop early spontaneous leiomyosarcoma tumours with occasional stabilization of p53 (ref. 58). Conversely, it has also been reported that repression of UBP43 shows antineoplastic effects in lung and kidney cancers and in acute promyelocytic leukemia^59^,^60^,^61^. In addition, UBE1L, the ISG15-activating E1 enzyme, has been shown to play a role in suppression of lung cancer and leukemia by promoting ISGylation of cyclin D1 and PML-RARα fusion protein, respectively^62^,^63^,^64^,^65^,^66^. Moreover, DNA-damaging agents, such as doxorubicin, were shown to induce ISGylation and caspase-2-mediated cleavage of ISGylated ΔNp63α, an anti-apoptotic and mitogenic oncoprotein, implicating the role of the ISG15 system in suppression of ΔNp63α-mediated tumorigenesis^32^. It has also been shown that ISGylation of PCNA plays a critical role in termination of ultraviolet-induced error-prone translesion synthesis and thus in the maintenance of genome stability for preventing DNA damage-induced tumorigenesis^33^. Finally, in this study, we have shown that DNA damage-induced p53 ISGylation inhibits cell growth and tumour development by promoting its transactivity. Thus, it appears that protein ISGylation plays a crucial role in positive regulation of DNA damage response, which in turn suppresses DNA damage-induced tumorigenesis.

## Methods

### Plasmids and antibodies

cDNAs for human ISG15 and UBCH8 and mouse UBP43 were subcloned into pFlag-CMV10 vector and/or pcDNA3-Myc vector^32^. Human p53 cDNAs were obtained from Korean human gene bank. p53, its deletion constructs, and K-to-R mutants were subcloned into pcDNA3-HA vector and pcDNA-HisMax vector (Invitrogen)^67^. Site-directed mutagenesis was performed as recommended by the manufacturer's instructions (Stratagene).

Antibodies used were mouse monoclonal anti-Xpress (Catalogue number P/N 46-0528, Invitrogen), mouse monoclonal anti-Flag M2 (F3165, Sigma), mouse monoclonal anti-Myc (9E10, Santa Cruz), mouse monoclonal anti-p53 (DO-1, Santa Cruz), rabbit polyclonal anti-p21 (C-19, Santa Cruz), mouse monoclonal anti-β-actin (C4, Santa Cruz), rabbit anti-phospho-p53 (9284S, Cell Signaling) and rabbit anti-acetyl-p53 (2525S, Cell Signaling). Polyclonal anti-ISG15 antibody was generated by injecting purified ISG15 protein to rabbit^32^. shRNA were purchased from Open Biosystems. Target sequences of shRNAs for ISG15 and EFP are: 5′- CTGAGCATCCTGGTGAGGAAT -3′ for ISG15; 5′- GAACTCATCTTTGCCAGTA -3′ (5′-UTR (untranslated region) for ISG15; 5′- GAGTGAGATCCAGACCTTGAA -3′ for EFP; 5′- GCAGAACTCTCCTTGGATA -3′ (3′-UTR) for EFP. All antibodies were diluted 1:1,000 with PBS containing 0.1% Triton X-100 and 3% BSA, except anti-β-actin, which was diluted 1:2,500.

### Immunoprecipitation and pull-down assay

For immunoprecipitation, cell lysates were prepared in buffer-A consisting of 50 mM Tris-HCl (pH 7.4), 150 mM NaCl, 1 mM EDTA, 1 mM NEM, 1 mM sodium orthovanadate, 1 mM NaF, 1 mM PMSF, 0.2% (v/v) Triton X-100 and 1X protease inhibitor cocktail (Roche). The cell lysates were incubated with appropriate antibodies for 1 h at 4 °C and then with protein A-conjugated Sepharose for the next 1 h. For Ni^2+^-NTA agarose (NTA: Qiagen) pull-down assay, the cell lysates were prepared in buffer-A containing 10 mM imidazole. After incubating cell lysates with NTA resin for 1 h at 4 °C, the samples were precipitated, washed three times with buffer-A containing 20 mM imidazole and subjected to SDS–polyacrylamide gel electrophoresis followed by immunoblot analysis. Supplementary Fig. 20 shows the full-blots of the data obtained from SDS–polyacrylamide gel electrophoresis.

### Cell culture and transfection

HEK293T, A549 and H1299 cells (from ATCC) were cultured at 37 °C in 5% CO_2_ atmosphere in DMEM supplemented with 10% FBS, 2 mM L-glutamine and 25 units ml^−1^ of penicillin and streptomycin. HCT116 cells were cultured as above, but using RPMI1640 medium in place of DMEM. Transfections of plasmids were performed using Metafectene (Biontex) and JetPEI (Polyplus) according to the manufacturer's instructions. All the cell lines were regularly tested for mycoplasma contamination.

### ISGylation assays

ISG15-conjugating system was overexpressed in HEK293T cells with HA- or HisMax-tagged p53. The cell lysates were prepared in buffer-A. The samples were incubated with appropriate antibodies for 2 h at 4 °C and then with protein A-conjugated Sepharose for the next 1 h. The precipitates were washed and subjected to immunoblot analysis.

### Luciferase assay

H1299 cells transfected with pcDNA-β-gal and *PG13-Luc*, *p21-Luc* or *BAX-Luc* were incubated for 24 h. After treatment with ultraviolet, the cell lysates were subjected to assay for the luciferase activity (Promega) as recommended by the manufacturer. Transfection efficiency was normalized by using β-galactosidase as a control.

### Cell growth and clonogenic assays

For cell growth assay, *p53*^*−/−*^ HCT116 cells (3.0 × 10^5^) were cultured in triplicates in 60 mm plates for 24 h. The cells were then treated with 0.1 μM doxorubicin for various periods before harvesting. Viable cells were counted following trypan blue staining. For clonogenic assay^68^, *p53*^*−/−*^ HCT116 cells that stably express either HisMax-tagged wild-type p53 or its 2KR mutant were plated in six-well plates at 500 cells in 2 ml of RPMI1640 medium per well. After incubation for 24 h, the cells were treated with 0.1 μM doxorubicin and further incubated for the next 10 days. The colonies developed were washed twice with PBS, fixed and stained with crystal violet for 20 min, washed twice with PBS and then counted.

### ChIP assay

HCT116 cells that had been treated with ultraviolet or doxorubicin were quickly washed with PBS, crosslinked in 1% formaldehyde for 10 min, collected by 100 mM Tris-HCl (pH 9.4) containing 10 mM DTT and incubated for 15 min at 30 °C. For chromatin fragmentation, the cells were sonicated in 50 mM Tris-HCl (pH 8.1) containing 1% SDS, 10 mM EDTA, 1X protease inhibitor cocktail. Proteins were immunoprecipitated in 20 mM Tris-HCl (pH 8.1) containing 1% Triton X-100, 2 mM EDTA, 150 mM NaCl and 1X protease inhibitor cocktail. Crosslinking was reversed by overnight incubation at 65 °C in 1% SDS and 0.1 M NaHCO_3_, and fragmented DNAs were purified by using QIAquick gel extraction kit (QIAGEN). Precipitated DNAs was then subjected to PCR (40 cycles) followed by electrophoresis or to quantitative PCR. The primers used for PCR analyses were listed in Supplementary Fig. 6.

### Quantitative RT-PCR

mRNA abundance was detected by using an ABI Prism 7,500 system and 2X PreMix SYBR Green (Enzynomics RT500S). Primer pairs were designed to amplify 50–200 bp mRNA-specific fragments, which were then confirmed as unique products by melting curve analysis. The PCR conditions were as follows: 95 °C (15 min) and 40 cycles of 95 °C (30 s), 60 °C (30 s), and 72 °C (30 s). The quantity of mRNA was calculated using the DDCt method and normalized to that of GAPDH. All the reactions were performed in triplicates. The primers used were 5′- GGTGGACAAATGCGACGAAC -3′ (forward) and 5′- ATGCTGGTGGAGGCCCTTAG -3′ (reverse) for ISG15; 5′- AGGTGGCCAAGAACTTGGTT -3′ (forward) and 5′- CACCACCTGGAAGTCCAACA -3′ (reverse) for UBE1L; 5′- GGAACCTGTCCAGCGATGAT -3′ (forward) and 5′- TCAGGTGGTAGGGAGGTTGGT -3′ (reverse) for UBCH8; 5′- GTGACCACGGCTTTGTCATCT -3′ (forward) and 5′- TAAAGTCCACCCTGAACTTATACATCAG -3′ (reverse) for EFP; 5′- GGAGGTGAAGGTCGGAGTCA -3′ (forward) and 5′- GACAAGCTTCCCGTTCTCAG -3′ (reverse) for GAPDH.

### *In vivo* tumorigenesis assays

*p53*^*+/+*^ HCT116 cells (5 × 10^6^) that stably express shNS or shISG15 and *p53*^*−/−*^ HCT116 cells (5 × 10^6^) that stably express wild-type p53 or its ISGylation-deficient KR mutant were subcutaneously injected into the upper thighs of 4-week-old BALB/c male nude mice. On the third day after injection, mice were intraperitoneally injected twice weekly with PBS or doxorubicin (1.25 mg kg^−1^). They were monitored regularly for tumour growth. Tumour volumes were calculated as (*a* × *a* × *b*)/2, in which *a* is the smallest diameter and *b* is the largest. Finally, the mice were killed and tumours were dissected out, photographed and subjected to immunoblot analysis.

### Animal study approval

All animal procedures were approved by the IACUC of Seoul National University, Korea.

### Statistics

All the values were given as mean±s.d. Statistical comparisons were made by two-tailed Student's *t*-test. *P* values less than 0.05 was considered statistically significant.

### Data availability

The authors declare that the data supporting the findings in this study are available within the article and its Supplementary Information files as well as upon request from the authors.

## Additional information

**How to cite this article:** Park, J. H. *et al*. Positive feedback regulation of p53 transactivity by DNA damage-induced ISG15 modification. *Nat. Commun.* 7:12513 doi: 10.1038/ncomms12513 (2016).

## Supplementary Material

Supplementary Information Supplementary Figures 1-20
