# Supplementary material for: Positive feedback regulation of p53 transactivity by DNA damage-induced ISG15 modification
Source: Nat Commun. 2016 Aug 22;7:12513. doi: 10.1038/ncomms12513 (PMC4996943; doi:10.1038/ncomms12513)

Supplementary figures

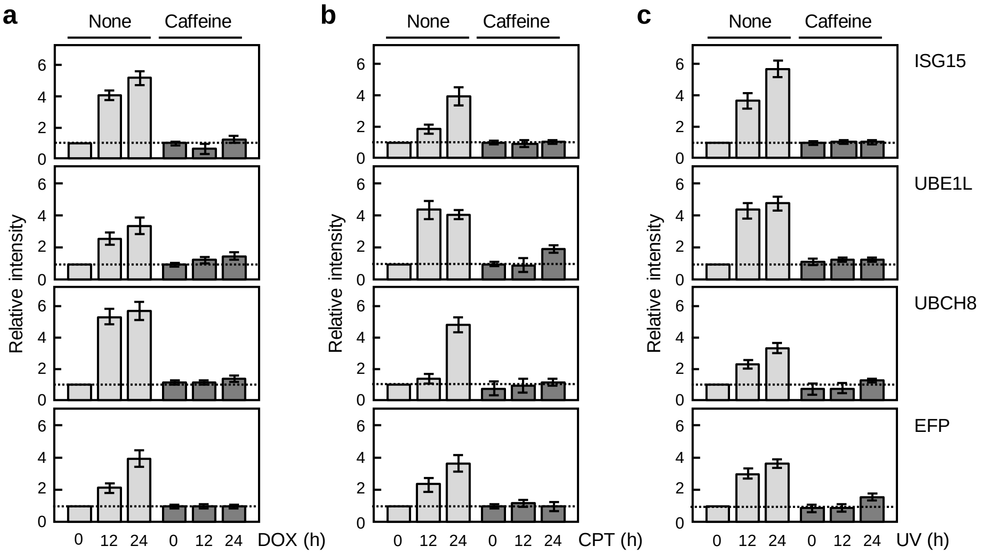

Supplementary Figure 1. Quantification of DNA damage-induced changes in the levels of ISG15-conjugating system. Experiments were performed as in Fig. 1 and the band intensities were determined by using a densitometer and normalized by those of β-actin. The normalized densities seen at ‘0’ time points were expressed as 1.0 and the others were as its relative values. Error bar, ± s.d. (*n*=3).

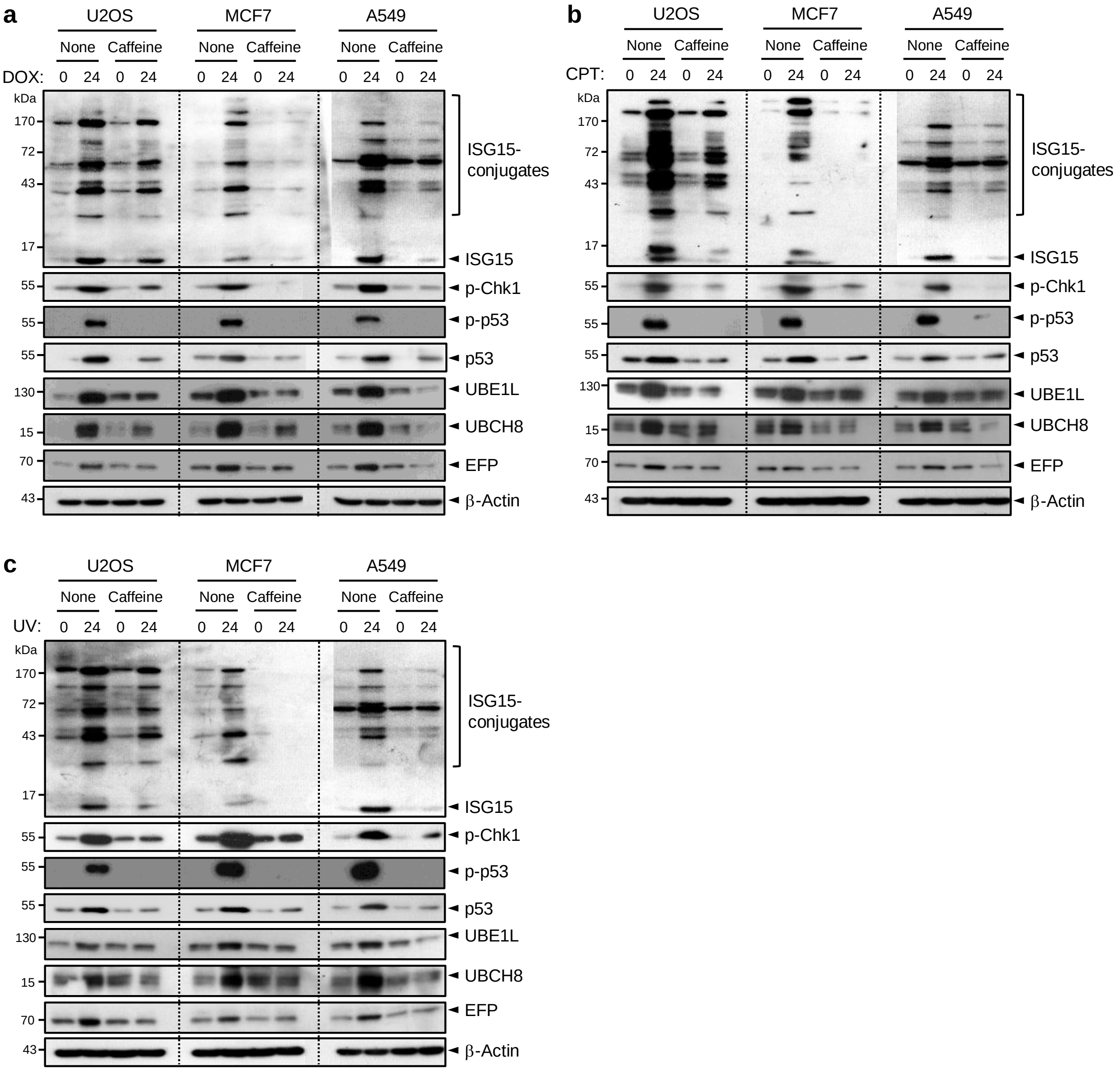

Supplementary Figure 2. Effect of caffeine on the expression of ISG15-conjugating system in various cancer cells. Doxorubicin (**a**), camptothecin (**b**), and UV (**c**) were treated to U2OS, MCF7, and A549 cells with and with caffeine, followed by immunoblot analysis as in Fig. 1.

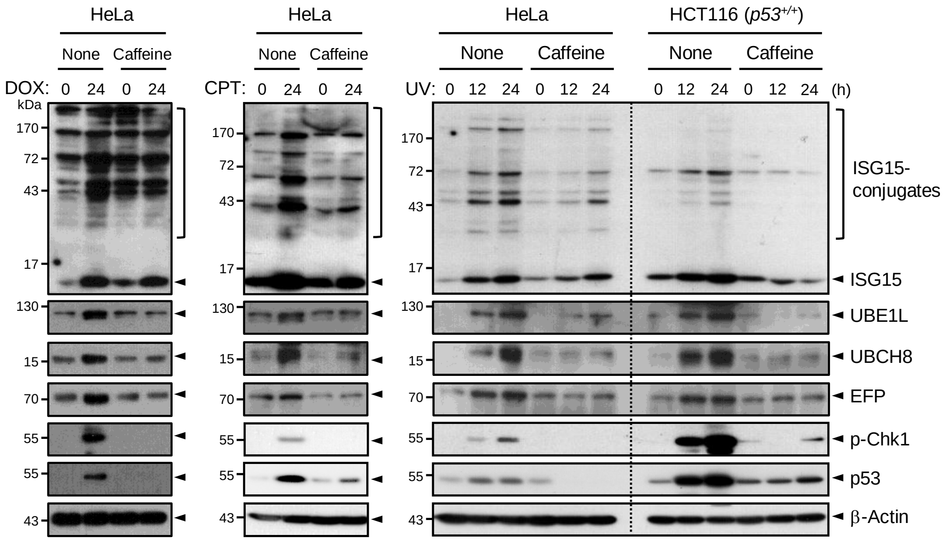

Supplementary Figure 3. Effect of caffeine on the expression of ISG15-conjugating system in HeLa cells. Experiments were performed as in Supplementary Fig. 1, but using HeLa cells.

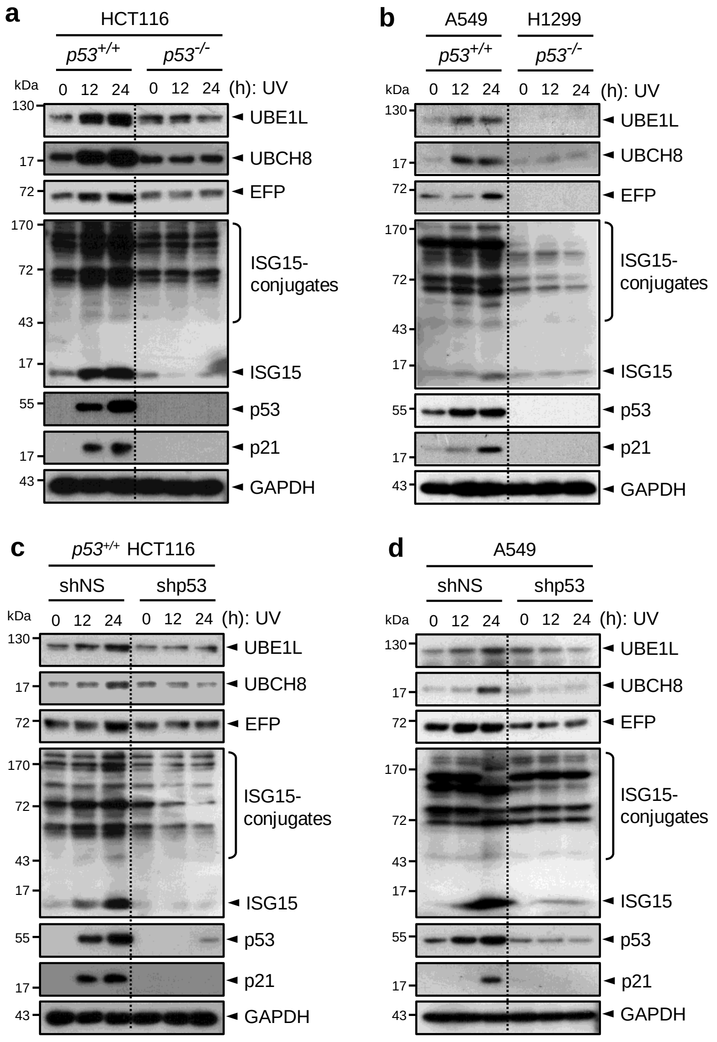

Supplementary Figure 4. p53 induces the expression of ISG15-conjugating system. After exposure to UV, HCT116 (*p53*$^{+/+}$ and *p53*$^{-/-}$) cells (**a**) and A549 (*p53*$^{+/+}$) and H1299 (*p53*$^{-/-}$) cells (**b**) were incubated for various periods. They were then subjected to immunoblot analysis. HCT116 (*p53*$^{+/+}$) cells (**c**) and A549 (*p53*$^{+/+}$) cells (**d**) that had been transfected with shNS or shp53 were exposed to UV. After incubation, they were subjected to immunoblot analysis.

```
a ISG15
RE1     GGACATGTGCTCCGTTCCTGCTACTTGTTT
RE2     TTCCAAGTTTACTAATTTTGCAAGTTG
RE3     GGGCATGCCT
RE3*    GGGACGTCCT

ISRE    GAAAGGGAAACCGAAACTGAAG
ISRE*   GAAAGGTCCACCTCCACTGAAG

b UBE1L
RE1     GGGCAAGGTGGGAGGATCACTTGAGGCCAGGCAT
RE2     GGCCAGGCATTCGAGACCAGCCTGGGCAAGATA
RE3     GAGCAAGCCT
RE1/2*  GGGACCTGTGGGAGGATCACTTGAGGCACTTCATTCGAGACCAGCCTGGGACCTATA

ISRE    CTTTCACTTTTCTTTTC
ISRE*   CTGGAACTTGGATTGGA

c UBCH8
RE1     GAACTAGAACCCAGGTCTCCCTGGGGCTTGTTC
RE2     CATCATGTTTCACAATACACTTGGAT
RE3     CAACAAGTCTCATCTTCCCCAGATGCTGCTTGGTG
RE4     CTGCAAGTACTCTGTCCTTGCAC
RE1*    GAAAGCTAACCCAGGTCTCCCTGGGGAGGTTTC

ISRE    AAAAGAGAAA
ISRE*   AACCTATCCA

d EFP
RE1     AAACAAGATGTGGGACAGGCCC
RE2     GGACAGGCCCATGGTGGCTCATGCCT
RE3     GGACTTGCTCCGAGCGCAAGTTT
RE1/2*  AAAACCTATGTGGGAACTTCCCATGGTGGCTACGTCCT

ISRE    TTTCGTTT
ISRE*   TGGAGTGG
```

Supplementary Figure 5. Sequences of *ISRE*s and putative *p53RE*s in the promoter regions of the genes for ISG15-conjugating system. Sequences of *ISRE*s and putative *p53RE*s in the promoter regions of the *ISG15* (**a**), *UBE1L* (**b**), *UBCH8* (**c**), and *EFP* genes (**d**) were shown. The asterisks indicate the mutant forms of *p53RE*s and *ISRE*s. The mutated nucleotides were indicated with the red letters.

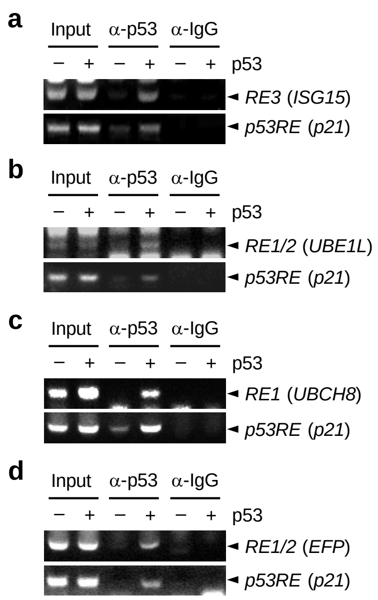

Supplementary Figure 6. Binding of p53 to *p53RE*s in the promoter regions of the *ISG15, UBE1L, UBCH8,* and *EFP* genes. HA-p53 was expressed in *p53*$^{-/-}$ HCT116 cells. Cell lysates were subjected to ChIP assay using anti-p53 antibody or anti-mouse IgG. Bound DNAs were subjected to PCR using the following probes for: (**a**) *ISG15*, 5'-TGCGCGATATTTAGGTGTTTCCAGGGTGTTGGGT-3' (forward) and 5'-CTGGTGGCCAAATTTGGCTTCAGTTTCGGTTTCC-3' (reverse); (**b**) *UBE1L*, 5'-CATGGTGGCTCACACCTGTAATCCCAGCAC-3' (forward) and 5'-ATGTTGCCTAGGCTGGTCTCGAATCTCTGG-3' (reverse); (**c**) *UBCH8*, 5'-AATGGTCCTGGCAAGCAGATAGGAGTTTCC-3' (forward) and 5'-CCACTGGTGTGCCTTTCATGTCCTTTCACA-3' (reverse); (**d**) *EFP*, 5'-TGGGCTGTGTGCCAATGTCCATTCTTAGAC-3' (forward) and 5'-ATACAAGCACGTGCCACCACACCCAGCTAA-3' (reverse); (A-D) *p21*, 5'-CAGGCTGTGGCTCTGATTGG-3' (forward) and 5'-TTCAGAGTAACAGGCTAAGG-3' (reverse).

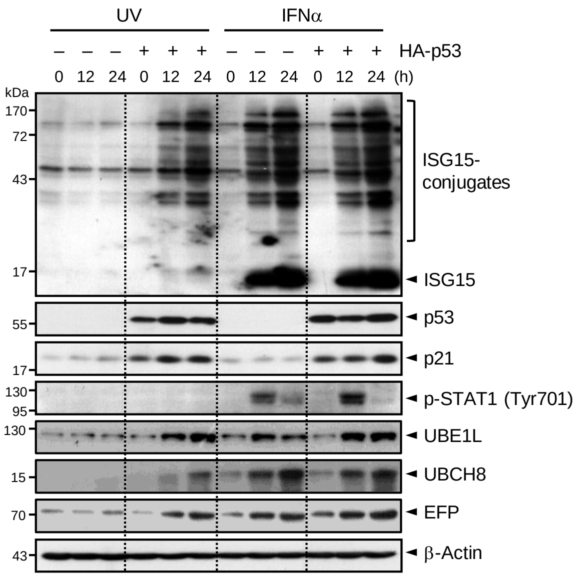

Supplementary Figure 7. UV and IFNα independently induce ISGylation of cellular proteins. *p53*$^{-/-}$ HCT116 cells that had been transfected with an empty vector (–) or pcDNA-HA-p53 (+) were exposed to UV and then incubated for increasing periods. The cells were also treated with IFNα for increasing periods. Cell lysates were subjected to immunoblot analysis.

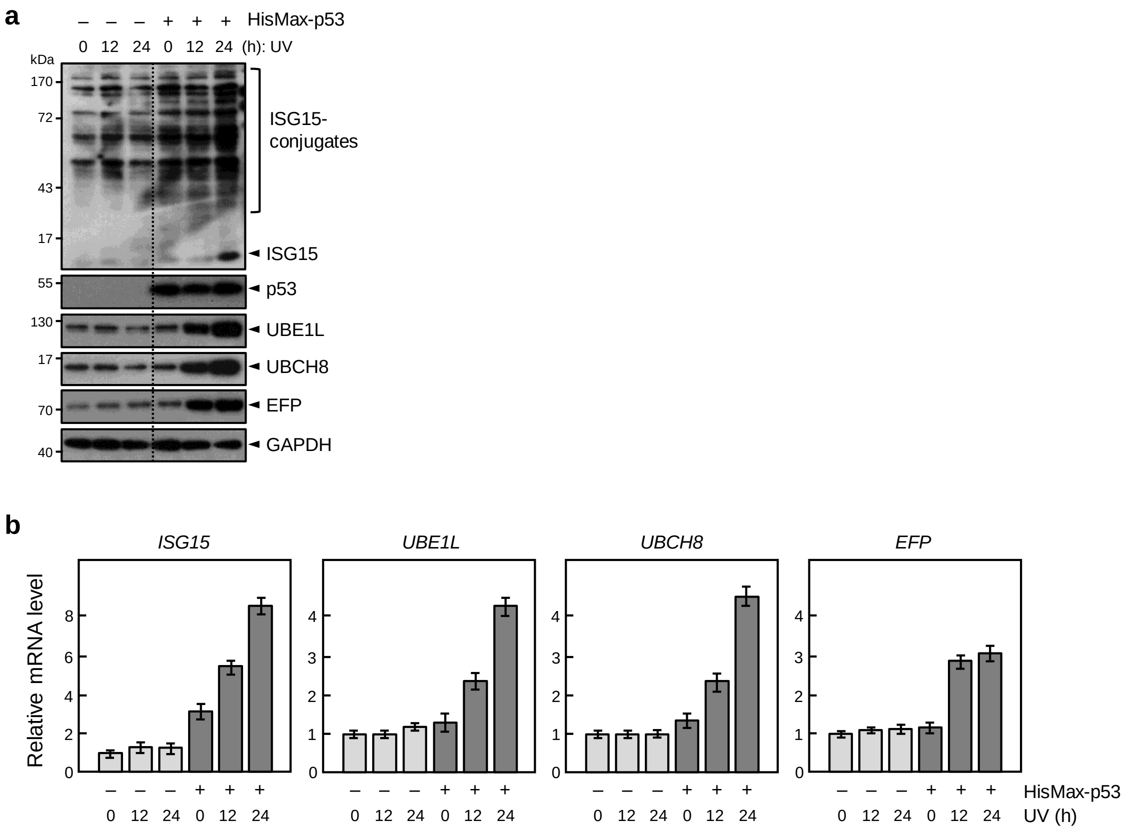

Supplementary Figure 8. p53 is required for UV-mediated increase in the expression of ISG15-conjugating system. (**a**) H1299 cells transfected with an empty vector (–) or pcDNA-HisMax-p53 (+) were exposed to UV followed by incubation for increasing periods. They were then subjected to immunoblot analysis. (**b**) The cells prepared as in **a** were subjected to qRT-PCR. The level of mRNA for each component of ISG15-conjugating system seen without ectopic expression of p53 at '0' time point was expressed as 1.0 and the others were as its relative values. Error bar, ± s.d. (*n*=3).

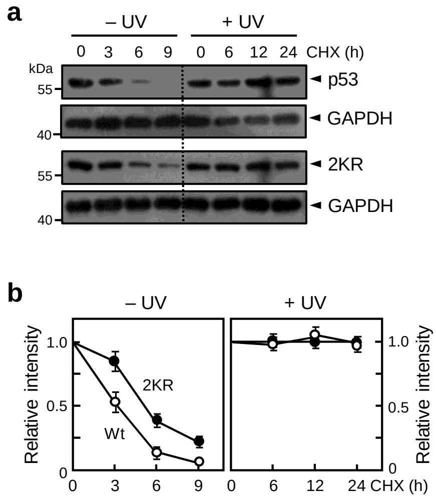

Supplementary Figure 9. Effect of ISGylation-deficient 2KR mutation on p53 stability. (**a**) p53 and 2KR were expressed in *p53*$^{-/-}$ HCT116 cells. After exposure to UV, cells were subjected to incubation with 0.2 mg/ml cycloheximide (CHX) for increasing periods followed by immunoblot analysis. (**b**) Experiments in **a** were repeated and the band intensities were scanned by using a densitometer and normalized by those of GAPDH. The normalized densities seen at '0' time points were expressed as 1.0 and the others were as its relative values. Error bar, ± s.d. (*n*=3).

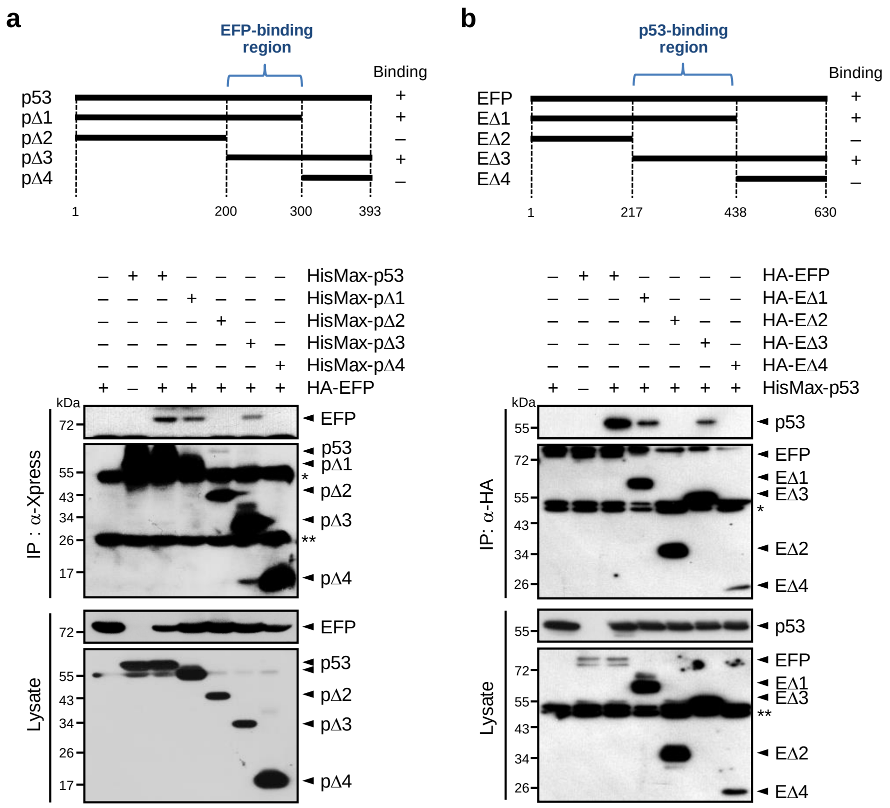

Supplementary Figure 10. Identification of the regions for interaction between p53 and EFP. (**a**) Deletions of p53 (pΔ1- pΔ4) were expressed in HEK293T cells with and without HA-EFP. Cell lysates were subjected to immunoprecipitation with anti-Xpress antibody followed by immunoblot with anti-HA or anti-Xpress antibody. They were also directly probed with anti-HA and anti-Xpress antibodies. The asterisk indicates the IgG heavy chain and the double asterisk shows the IgG light chain. (**b**) Deletions of EFP (EΔ1-EΔ4) were expressed in HEK293T cells with and without HisMax-p53. Cell lysates were subjected to immunoprecipitation with anti-HA antibody followed by immunoblot with anti-Xpress or anti-HA antibody. They were also directly probed with anti-Xpress and anti-HA antibodies. The asterisk indicates the IgG heavy chain and the double asterisk shows a nonspecific band.

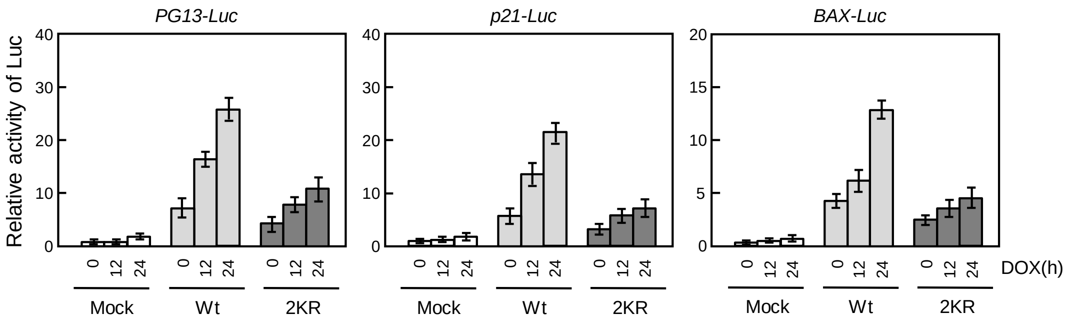

Supplementary Figure 11. ISGylation promotes p53 transactivity. Experiments were performed as in Fig. 6a, but in the presence of doxorubicin in place of UV exposure. The luciferase activity seen without any treatment (i.e., ‘0’ time in Mock) was expressed as 1.0 and the others were as its relative values.

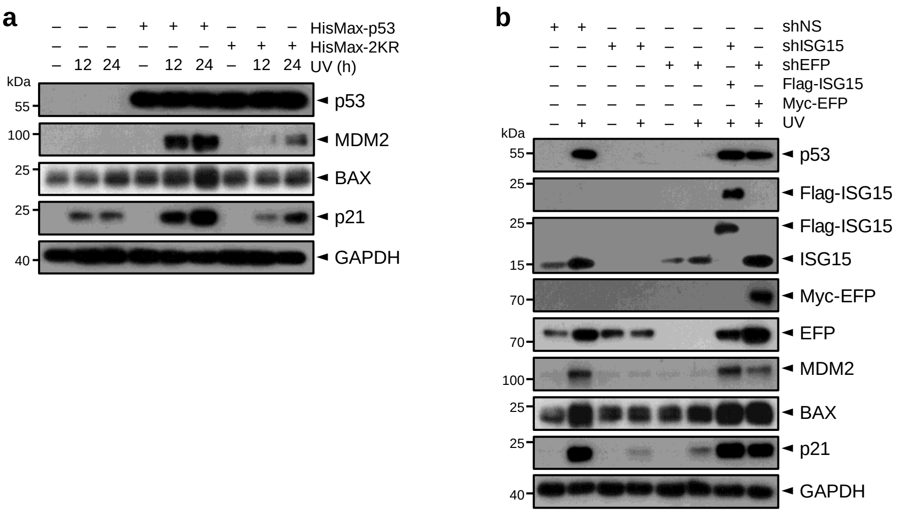

Supplementary Figure 12. Immunoblot analysis of cells used for determining p53 transactivity. Cells prepared as in Fig. 6a (**a**) and 6b (**b**) were subjected to immunoblot analysis.

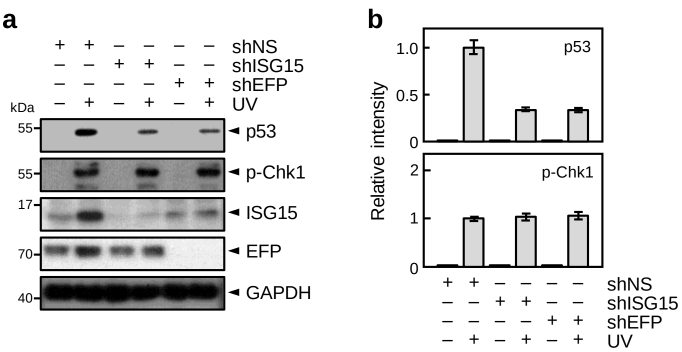

Supplementary Figure 13. Effect of knockdown of ISG15 or EFP on UV-induced Chk1 phosphorylation. (**a**) HCT116 (*p53*$^{+/+}$) cells transfected with shNS, shISG15, or shEFP were exposed UV. They were then subjected to immunoblot analysis. (**b**) Experiments were performed as in **a** and the band intensities were determined by using a densitometer and normalized by those of GAPDH. The normalized densities for p53 and p-Chk1 seen in the presence of shNS and UV were expressed as 1.0 and the others were as its relative values. Error bar, ± s.d. (*n*=3).

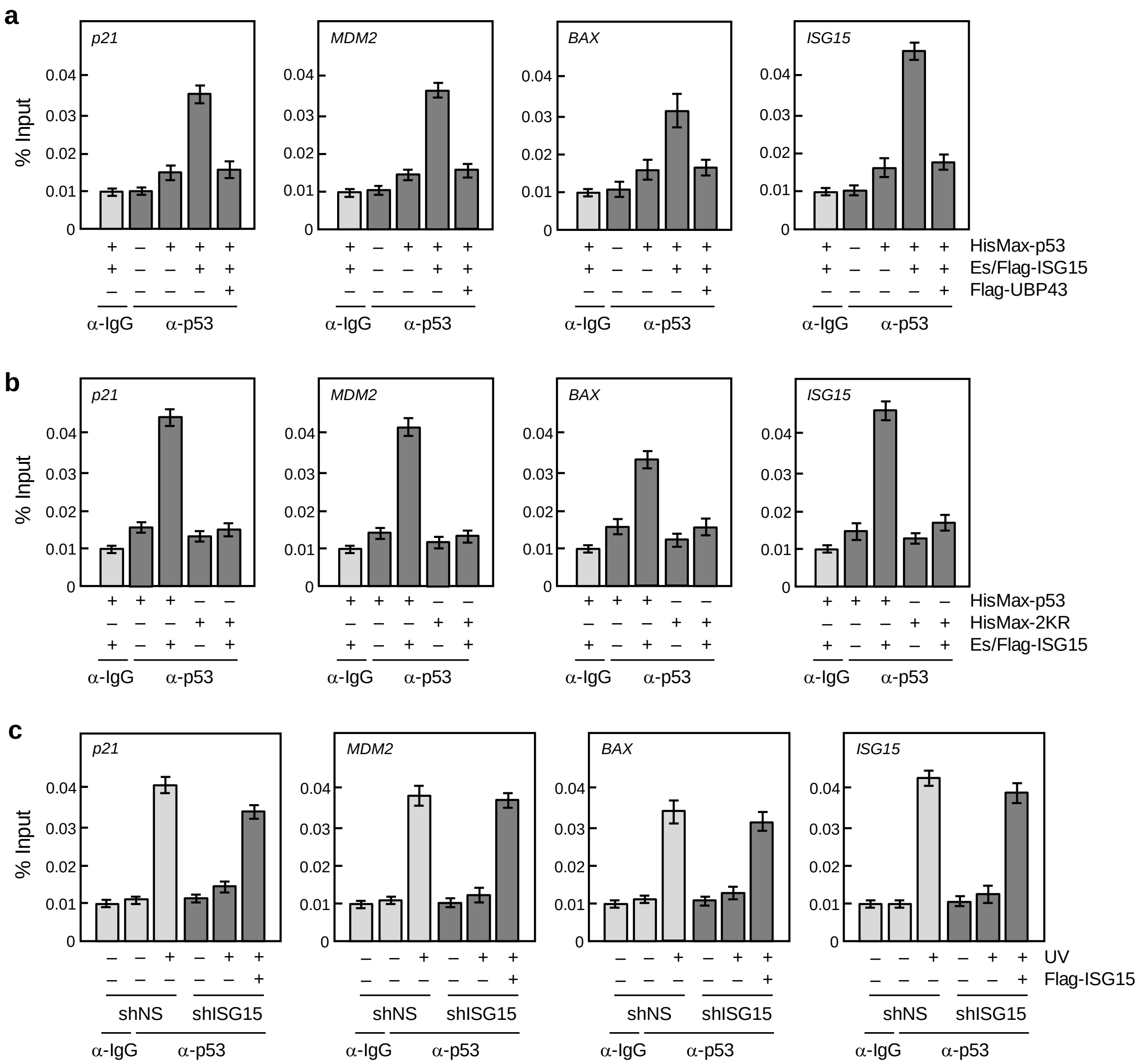

Supplementary Figure 14. Recruitment of p53 to *p53RE*s of the *p21*, *MDM2*, *BAX*, and *ISG15* genes. (**a-c**) Experiments were performed as in Fig. 6c-e, respectively, but the bound DNAs were subjected to qPCR using the probes for *p53RE*s of *CDKN1*, *MDM2*, *BAX*, and *ISG15*. Error bar, ± s.d. (*n*=4).

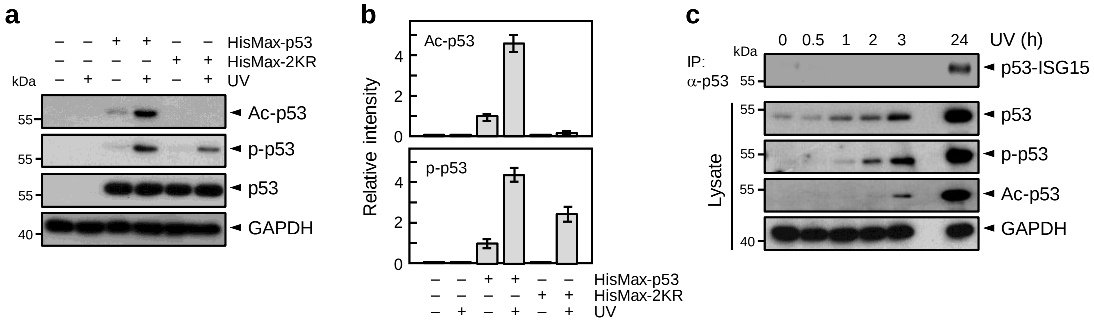

Supplementary Figure 15. ISGylation of p53 promotes its acetylation and phosphorylation. (**a**) HisMax-tagged p53 or 2KR was expressed H1299 cells. After exposure to UV, cells were subjected to incubation for 24 h followed by immunoblot analysis. (**b**) Experiments were performed as in **a** and the band intensities were determined by using a densitometer and normalized by those of GAPDH. The normalized densities for Ac-p53 and p-p53 seen with HisMax-p53 and without UV treatment were expressed as 1.0 and the others were as its relative values. Error bar, ± s.d. (*n*=3). (**c**) After UV treatment, *p53*$^{+/+}$ HCT116 cells were incubated for increasing periods. Cell lysates were subjected to immunoprecipitation with anti-p53 antibody followed by immunoblot with anti-ISG15 antibody. They were also directly subjected to immunoblot analysis.

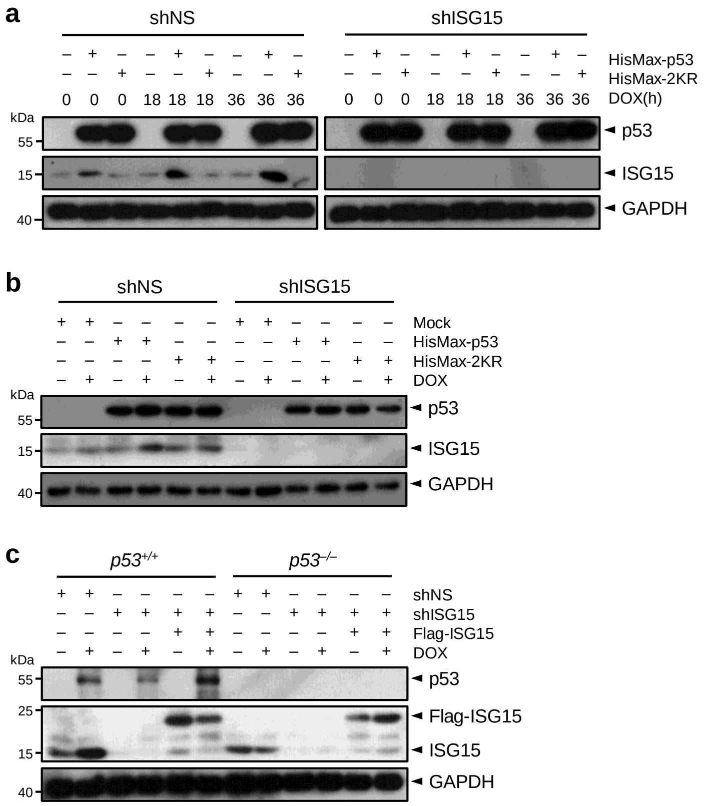

Supplementary Figure 16. Immunoblot analysis of cells used for determining cell growth and colony formation. Cells prepared as in Fig. 7a (**a**), 7b (**b**) , and 7d (**c**) were subjected to immunoblot analysis.

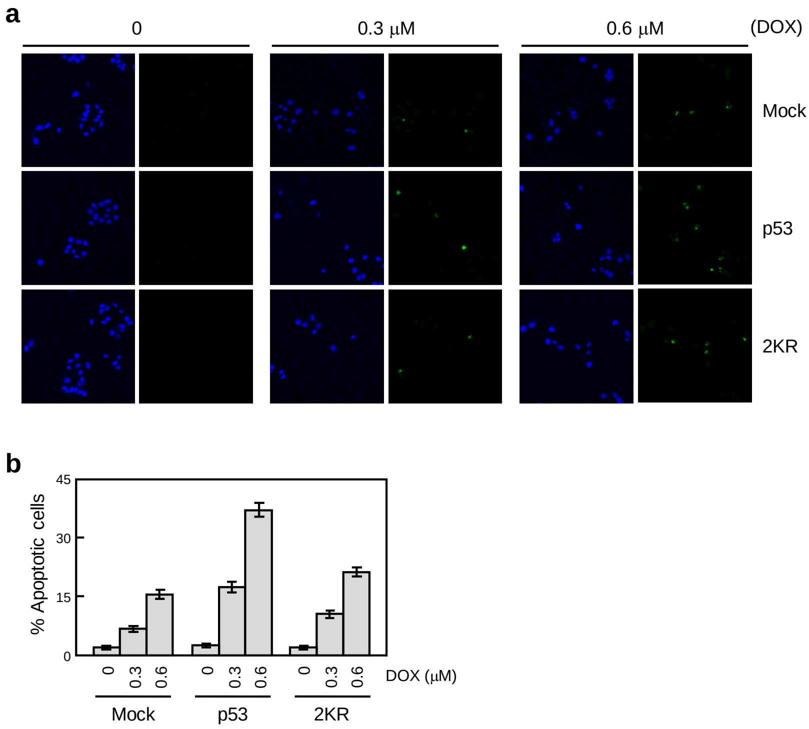

Supplementary Figure 17. Promotion of doxorubicin-induced apoptosis by p53 ISGylation. (**a**) *p53*$^{-/-}$ HCT116 cells expressing HisMax-tagged p53, its 2KR mutant, or an empty vector (Mock) were treated with different concentrations of doxorubicin. They were then subjected to TUNEL assay. (**b**) Experiments were performed as in **a** and the number of TUNEL-positive cells were counted and expressed as percentage of total number of cells. Error bar, ± s.d. (*n*=3).

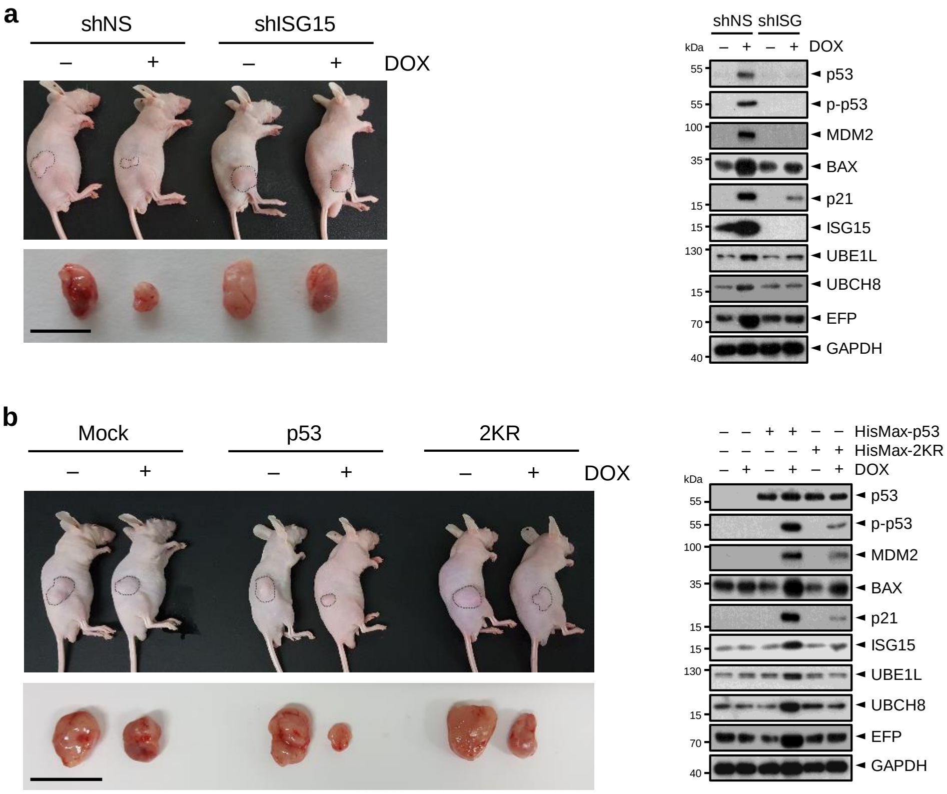

Supplementary Figure 18. ISGylation of p53 suppresses tumor growth. *p53*$^{+/+}$ HCT116 cells expressing shNS or shISG15 (**a**) and *p53*$^{-/-}$ HCT116 cells expressing wild-type p53 or its ISGylation-deficient 2KR mutant (**b**) were injected to BALB/c nude mice. Mock in **b** indicates mice injected with cells that had been transfected with an empty vector. After treatment with PBS or doxorubicin, tumors were dissected out and photographed (left panels). Lysates were prepared from the tumor tissues, and subjected to immunoblot analysis (right panels). Bar, 20 mm.

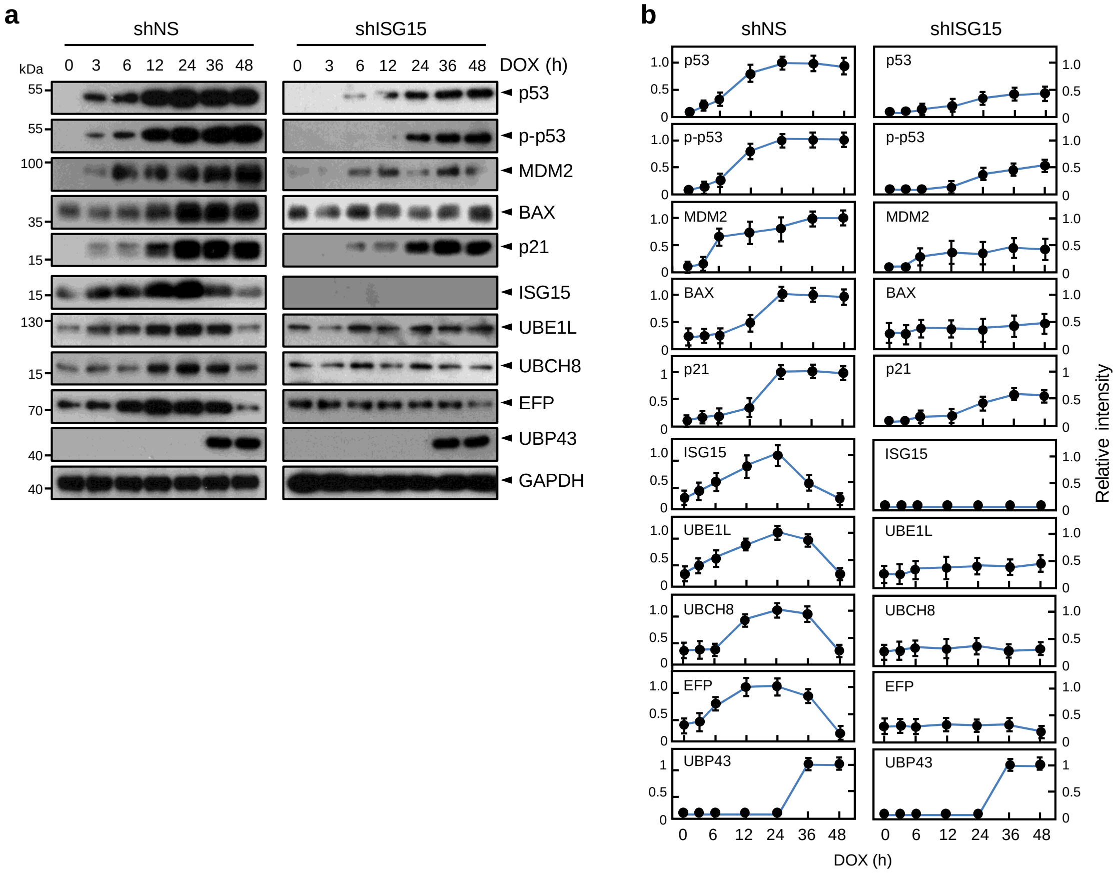

Supplementary Figure 19. UBP43 represses p53-induced expression of ISG15-conjugating system. (**a**) *p53*$^{+/+}$ HCT116 cells that stably express shNS or shISG15 were treated with doxorubicin for increasing periods. They were then subjected to immunoblot analysis. (**b**) Experiments were performed as in **a** and the band intensities were determined by using a densitometer and normalized by those of GAPDH. The normalized peak densities of the indicated proteins seen with shNS were expressed as 1.0 and the others were as its relative values. Error bar, ± s.d. (*n*=3).

Fig. 1a

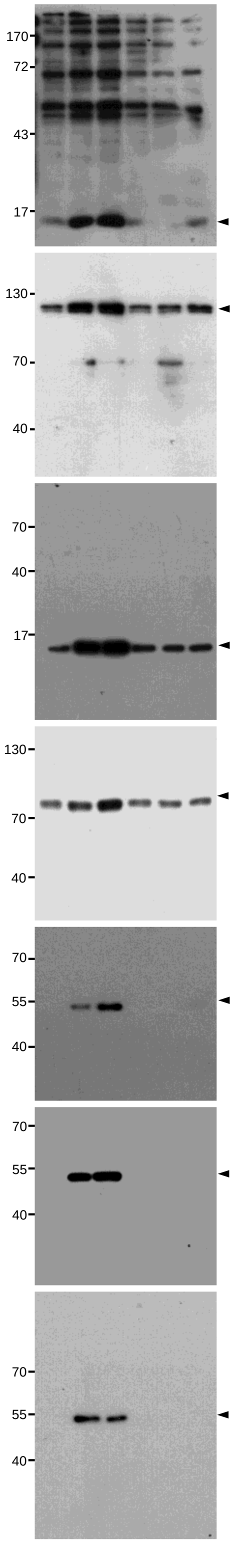

Fig. 1b

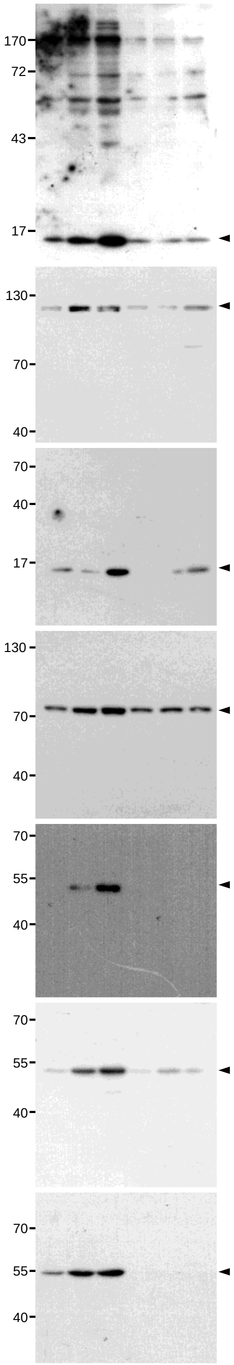

Fig. 1c

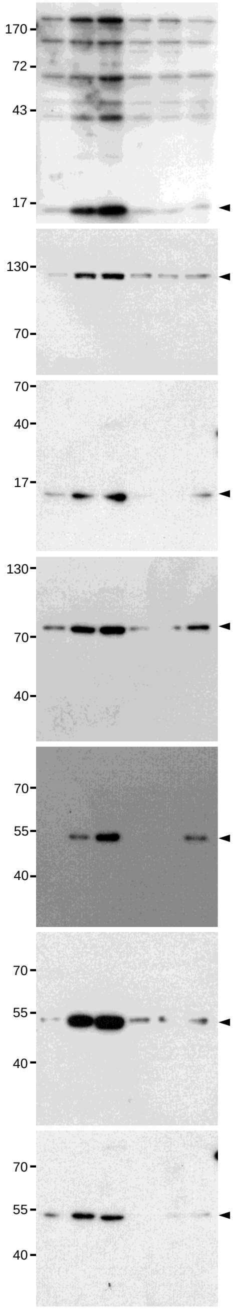

Fig. 4b

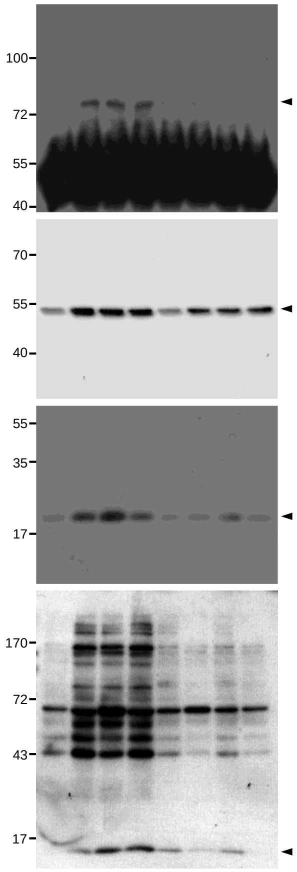

Supplementary Figure 20. Full blots.

Fig. 4d

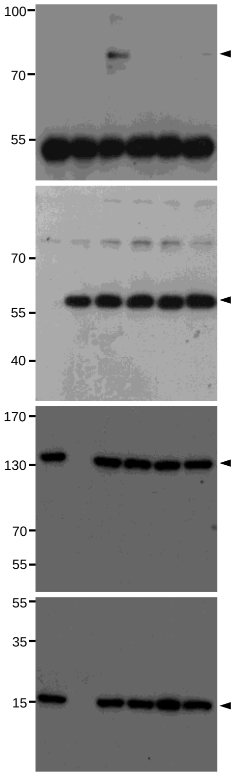

Fig. 4e

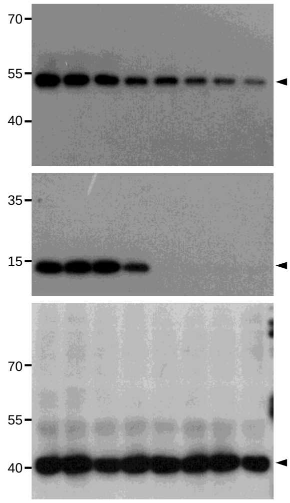

Fig. 5b

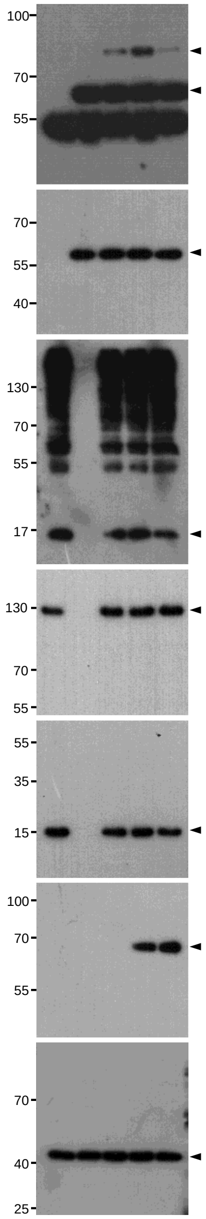

Fig. 5c

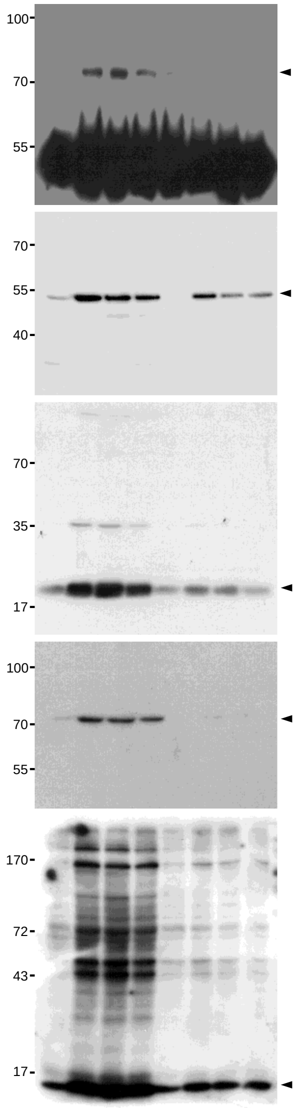

Fig. 5d

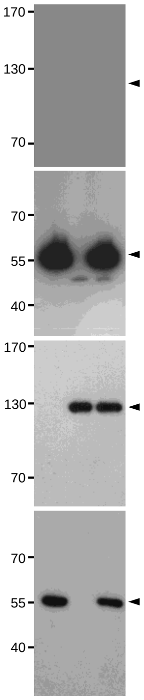

Fig. 5e

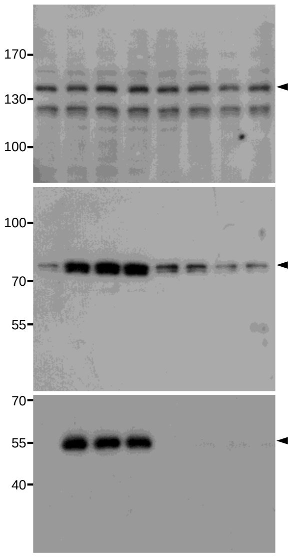

Fig. 5f

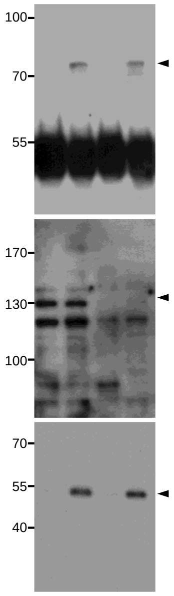

Fig. 6e

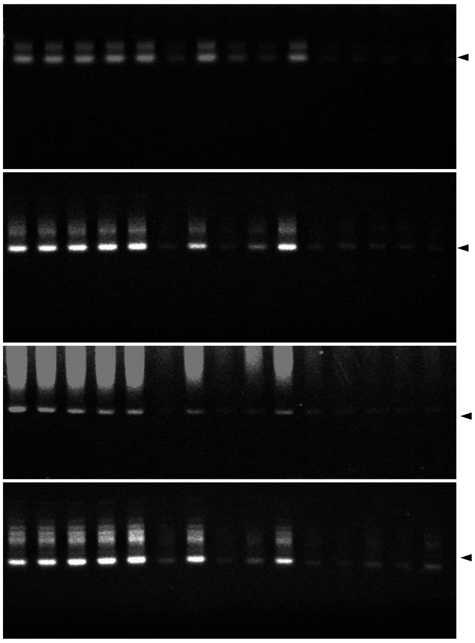

Fig. 6c

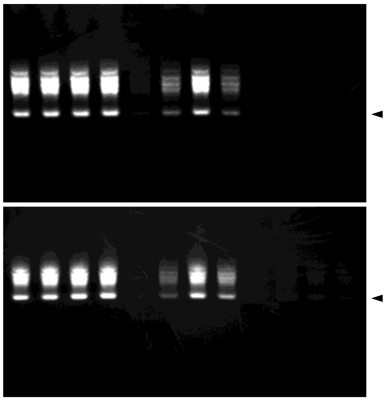

Fig. 6d

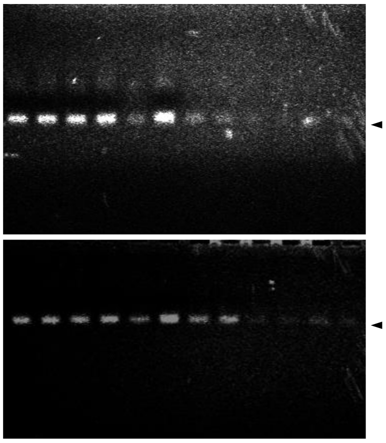

S. Fig. 2a

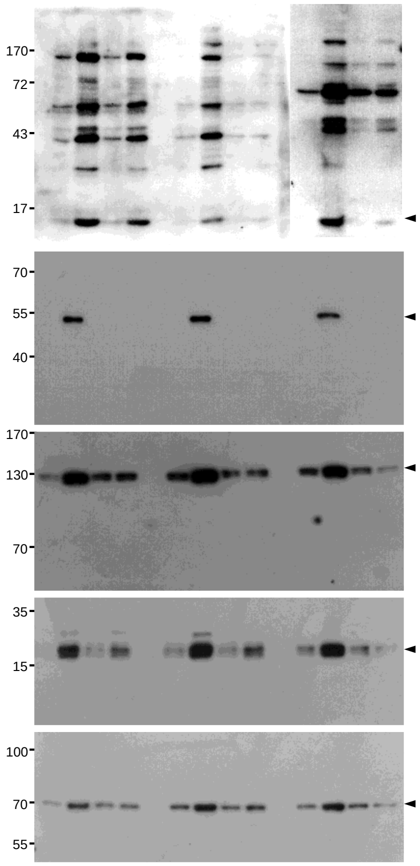

S. Fig. 2b

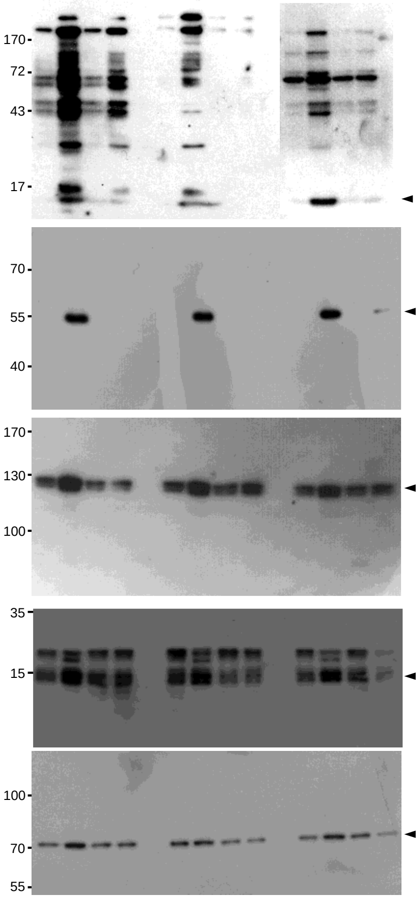

S. Fig. 2c

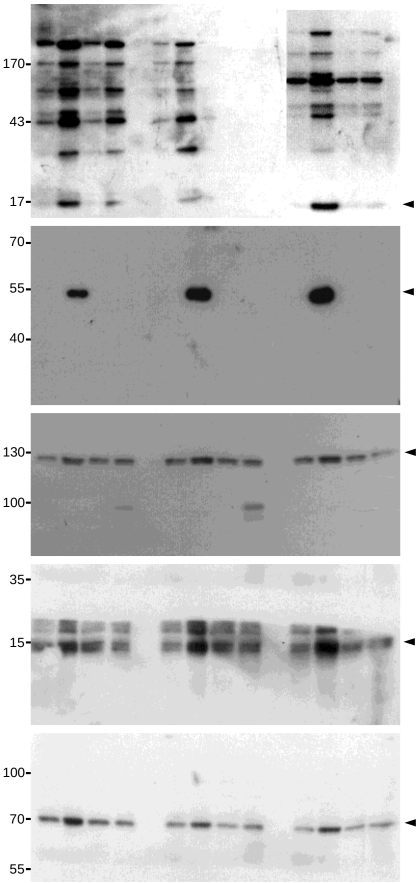

S. Fig. 3

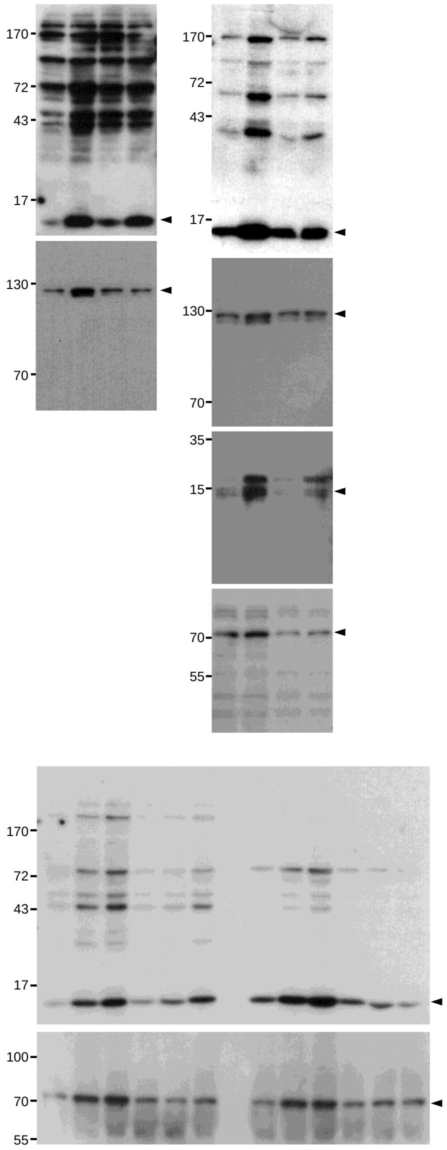

S. Fig. 4a

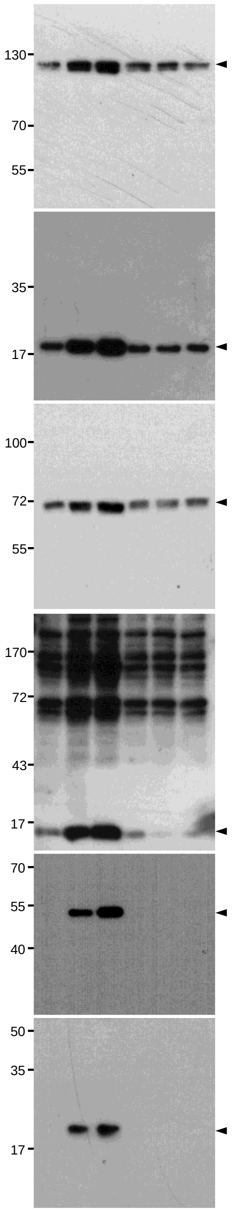

S. Fig. 4c

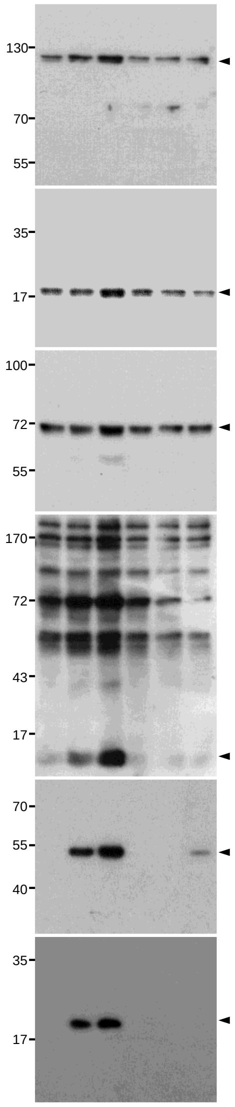

S. Fig. 8a

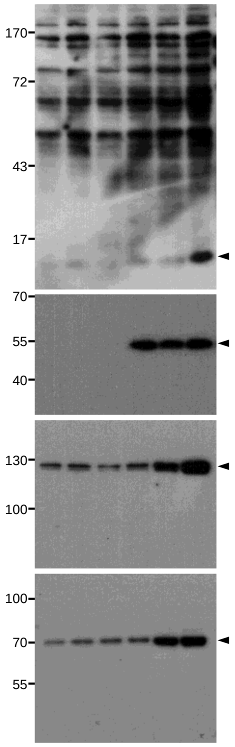

S. Fig. 9a

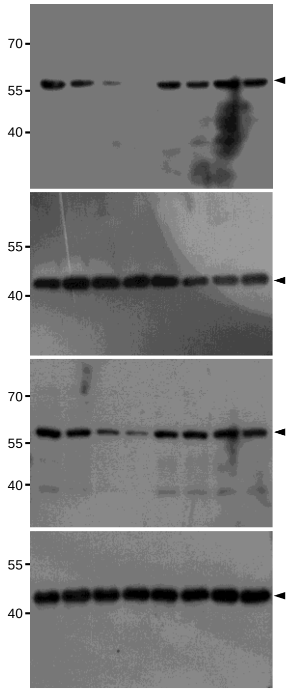

S. Fig. 13a

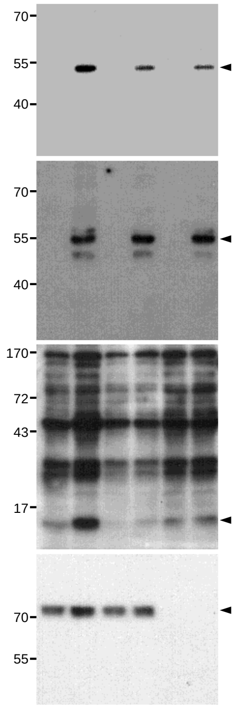

S. Fig. 15a

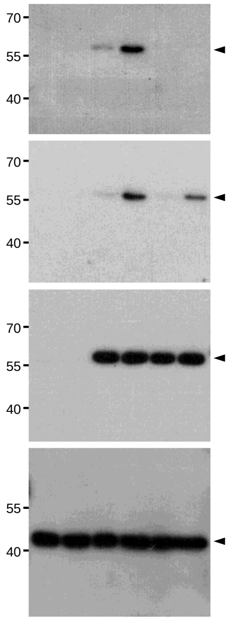

S. Fig. 15c

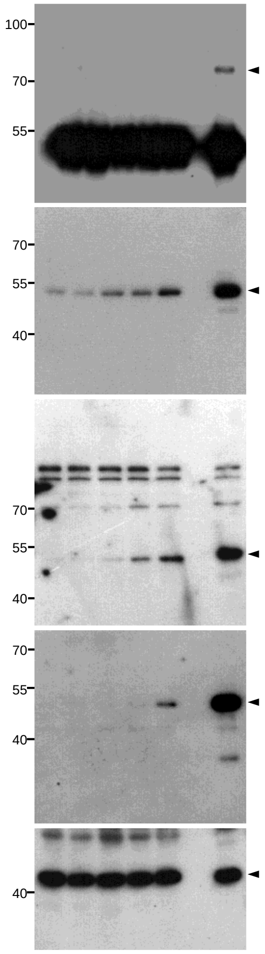

S. Fig. 16a

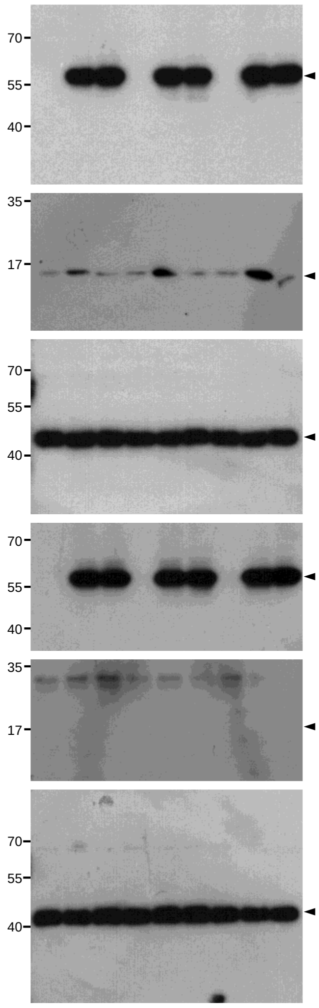

S. Fig. 16b

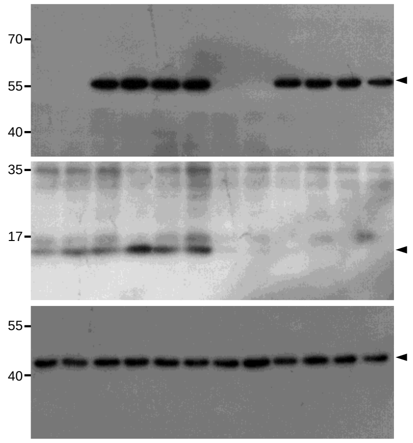

S. Fig. 18a

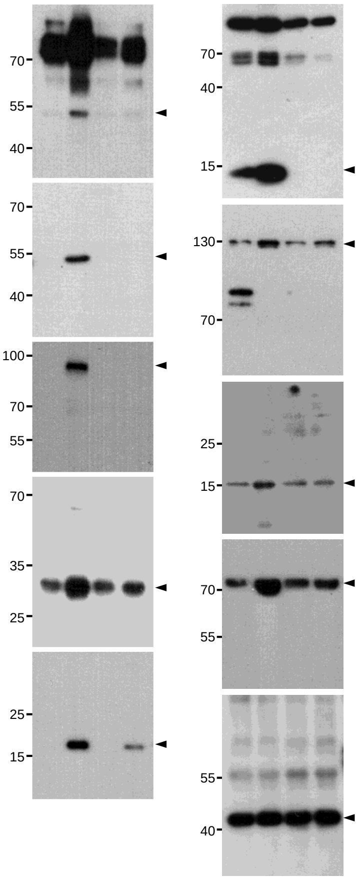

S. Fig. 19a

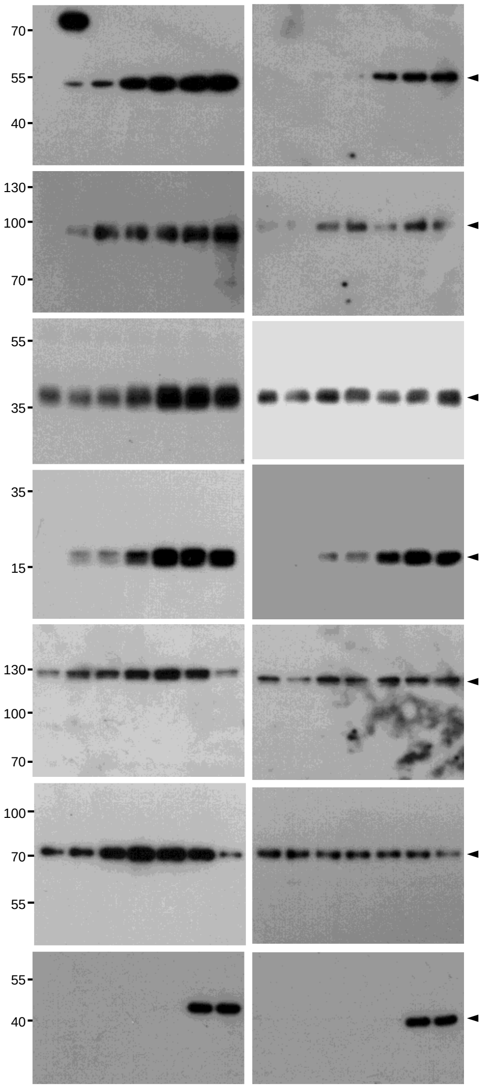

Supplement: Supplementary Information — Supplementary Figures 1-20 [file ncomms12513-s1.pdf]
